# MUSE-XAE: MUtational Signature Extraction with eXplainable AutoEncoder enhances tumour types classification

**DOI:** 10.1093/bioinformatics/btae320

**Published:** 2024-05-16

**Authors:** Corrado Pancotti, Cesare Rollo, Francesco Codicè, Giovanni Birolo, Piero Fariselli, Tiziana Sanavia

**Affiliations:** Computational Biomedicine Unit, Department of Medical Sciences, University of Torino, via Santena 19, Torino 10126, Italy; Computational Biomedicine Unit, Department of Medical Sciences, University of Torino, via Santena 19, Torino 10126, Italy; Computational Biomedicine Unit, Department of Medical Sciences, University of Torino, via Santena 19, Torino 10126, Italy; Computational Biomedicine Unit, Department of Medical Sciences, University of Torino, via Santena 19, Torino 10126, Italy; Computational Biomedicine Unit, Department of Medical Sciences, University of Torino, via Santena 19, Torino 10126, Italy; Computational Biomedicine Unit, Department of Medical Sciences, University of Torino, via Santena 19, Torino 10126, Italy

## Abstract

**Motivation:**

Mutational signatures are a critical component in deciphering the genetic alterations that underlie cancer development and have become a valuable resource to understand the genomic changes during tumorigenesis. Therefore, it is essential to employ precise and accurate methods for their extraction to ensure that the underlying patterns are reliably identified and can be effectively utilized in new strategies for diagnosis, prognosis, and treatment of cancer patients.

**Results:**

We present MUSE-XAE, a novel method for mutational signature extraction from cancer genomes using an explainable autoencoder. Our approach employs a hybrid architecture consisting of a nonlinear encoder that can capture nonlinear interactions among features, and a linear decoder which ensures the interpretability of the active signatures. We evaluated and compared MUSE-XAE with other available tools on both synthetic and real cancer datasets and demonstrated that it achieves superior performance in terms of precision and sensitivity in recovering mutational signature profiles. MUSE-XAE extracts highly discriminative mutational signature profiles by enhancing the classification of primary tumour types and subtypes in real world settings. This approach could facilitate further research in this area, with neural networks playing a critical role in advancing our understanding of cancer genomics.

**Availability and implementation:**

MUSE-XAE software is freely available at https://github.com/compbiomed-unito/MUSE-XAE.

## 1 Introduction

Mutational signatures are patterns of somatic mutations that reflect the underlying biological processes driving the cancer development (Alexandrov *et al.* 2013a, 2013b, Helleday *et al.* 2014). Mutational signatures have become a valuable resource for deciphering the genetic alterations underpinning cancer and for developing targeted therapies (Schulze *et al.* 2015, Secrier *et al.* 2016, Ma *et al.* 2018). In recent years, many methods have been developed for the extraction of mutational signatures, most of which are based on matrix factorization techniques, such as non-negative matrix factorization (NMF) (Ardin *et al.* 2016, Blokzijl *et al.* 2018, Bayati *et al.* 2020, Degasperi *et al.* 2020, Vöhringer *et al.* 2021, Islam *et al.* 2022) and its probabilistic versions (Fischer *et al.* 2013, Rosales *et al.* 2017, Gori and Baez-Ortega 2018). These approaches have been applied to several types of cancer, identifying more than 60 distinct signatures associated with specific mutational processes (Tate *et al.* 2019, Alexandrov *et al.* 2020).

Although these techniques have proven to be highly effective for the extraction of mutational signatures, some studies have highlighted possible issues that may arise during the extraction and should deserve attention and further investigation (Maura *et al.* 2019, Koh *et al.* 2021). Many of the mutational signatures found in the COSMIC catalogue currently have no known aetiology. Some are merely statistically linked to a specific process, whereas others lack any statistical association. In addition, the high degree of similarity between certain signatures may suggest the existence of nonbiological, overfitted signals (Schumann *et al.* 2019, Lal *et al.* 2021, Pancotti *et al.* 2022).

Future extraction techniques should consider these issues, implementing constraints in the decomposition to reduce the detection of overly similar profiles. Furthermore, NMF identifies only linear interactions, which could be a limitation as it might not capture potential nonlinear dependencies within the genome that can contribute to cancer development (Wojtowicz *et al.* 2021, Kičiatovas *et al.* 2022).

To overcome these challenges, we present MUSE-XAE, MUtational Signature Extraction with eXplainable AutoEncoder. This model includes a nonlinear encoder and a linear decoder with a non-negative constraint and a minimum volume regularization (Miao and Qi 2007) to detect potential nonlinear dependencies while preserving signature interpretability. Autoencoders have been successfully implemented in various domains, including genomics, to obtain compact and informative data representations. Autoencoders employing a hybrid architecture with a nonlinear encoder and a linear decoder have been applied in the context of single-cell RNA-seq and transcriptomic data (Svensson *et al.* 2020, Seninge *et al.* 2021), achieving great success due to their explainability while preserving powerful performance capabilities. However, to the best of our knowledge, no applications of this architecture have been developed so far in the context of mutational signature analysis.

To fill this gap, this paper introduces MUSE-XAE and shows its effectiveness on different cancer datasets by comparing it to existing state-of-the-art approaches in both synthetic scenarios and real-world applications. In a comprehensive comparison with 10 other *de novo* extraction tools considering realistic synthetic scenarios, MUSE-XAE resulted the best performing model with high sensitivity and precision in recovering the true signature profiles. We then evaluated our approach in two real world settings: 2780 cancer samples from the Pan-Cancer Analysis of Whole Genomes (PCAWG) study (ICGC 2020), and 1865 samples from another whole-genome sequencing (WGS) cohort (Islam *et al.* 2022). MUSE-XAE was able to extract highly discriminative signature profiles that can significantly improve the classification of tumour types and subtypes.

## 2 Materials and methods

### 2.1 MUSE-XAE architecture

An autoencoder is a type of neural network capable of learning a lower-dimensional representation of the data. Given an input space *X*, it consists of an encoder network *f*, represented by one or more layers, that maps the input data to a lower-dimensional latent space *Z*, and a decoder network *g* that reconstructs the input space from the latent representation. The goal of an autoencoder is to minimize the reconstruction error $L(x,\hat{x})$ between the original input *x* and the reconstructed output $\hat{x}$. The general equations that define an autoencoder are:

(1) z=f(x)=Encoder

(2) $$\hat{x}=g(z)=g(f(x))=\mathrm{Decoder}$$

usually, both *f* and *g* represent nonlinear activation functions.

**Figure 1.**
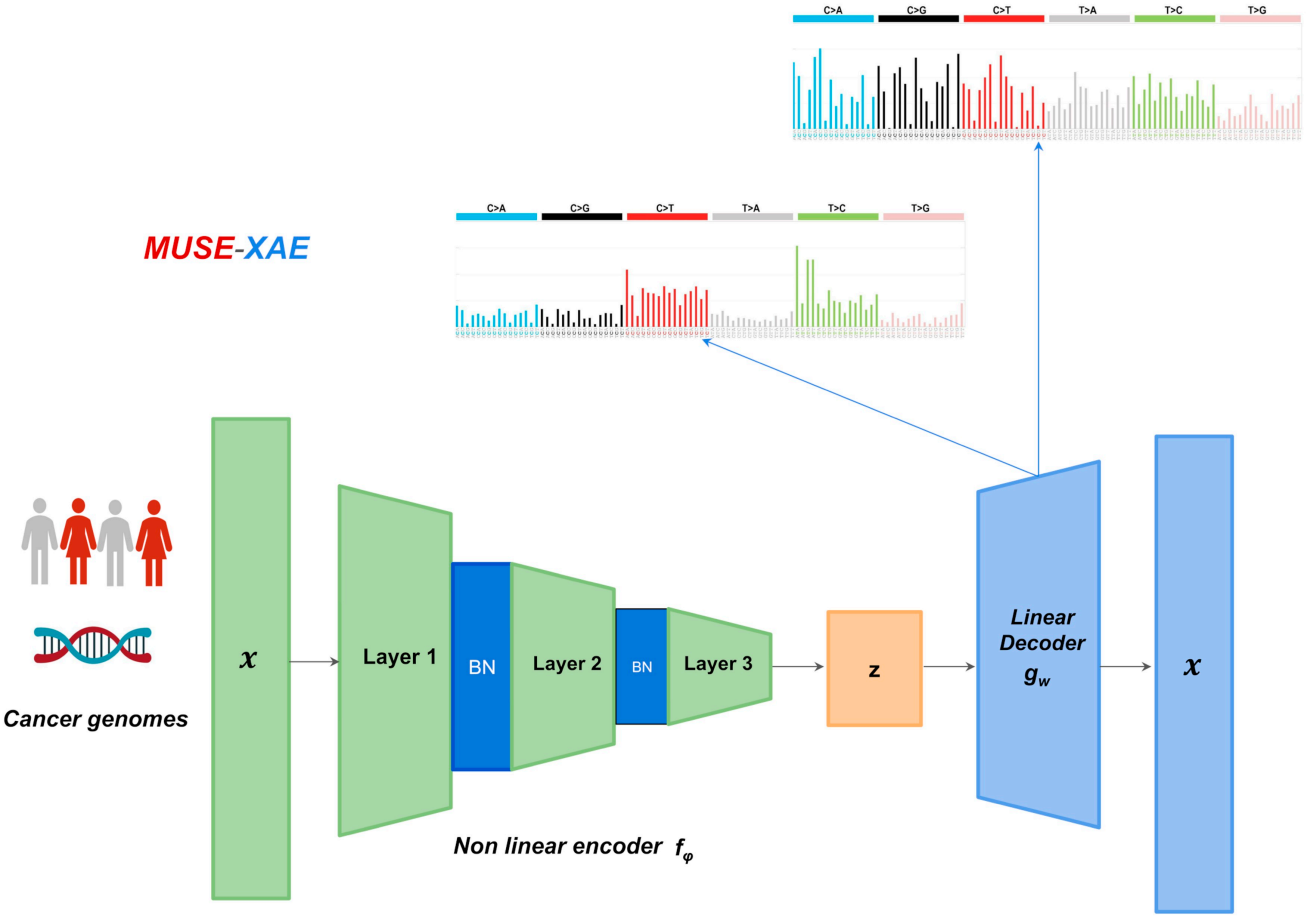
MUSE-XAE schematic architecture. MUSE-XAE features a nonlinear encoder, made up of three layers that leverage a softplus activation function with batch normalization. The decoder is designed to be linear to enhance the interpretability.

MUSE-XAE implements a hybrid architecture with a nonlinear encoder to learn a latent representation *z* of cancer samples, and a linear decoder with a non-negative constraint and minimum volume regularization to reconstruct the original input, such as $\hat{x}=zW^{T}$. Specifically, MUSE-XAE encoder *f* includes three hidden layers with batch normalization and a softplus activation function. The latter function offers continuous differentiability and a smoother transition from negative to positive values compared to ReLU, reducing the risk of neuron inactivation, with improved stability (Zheng *et al.* 2015). The decoder *g* is characterized by a weight matrix *W* with non-negativity constraint, a linear activation function that ensures interpretability, and a minimum volume regularization that helps the model find a more disentangled representation. In addition, MUSE-XAE exploits a non-negative Poisson likelihood function to take into consideration the count nature of the input data, and an early stopping criterion to avoid overfitting. Considering all the contributions, the total loss function $L(x,\hat{x})$ can be written as:

(3) $$\begin{array}{c} L(x,\hat{x})=-x\;\text{log}(\hat{x})\;+\;\hat{x}\;+\;\beta \;\text{log}\;\left(\det\left(WW^{T}\;+\;I\right)\right)\\ \begin{array}{c} \text{subjected to}\;W\geq 0 \end{array} \end{array}$$

where the first two terms refer to the Poisson likelihood function, while the third term represents the logarithm of the minimum volume constraint. The β coefficient regulates the strength of the regularization. Referring to the mutational signature terminology, the latent representation *z* represents the cancer genome’s exposures, while the decoder weight matrix *W* represents the mutational signatures. MUSE-XAE architecture is displayed in Fig. 1.

### 2.2 Signatures extraction

MUSE-XAE *de novo* extraction procedure is summarized in Algorithm 1. Training a neural network requires a substantial amount of data to exploit its capacity and fit the parameters effectively. In MUSE-XAE implementation, we used a data augmentation strategy to overcome this challenge.

Algorithm 1. MUSE-XAE *De Novo* Extraction procedure

Given $C\in R^{m\times 96}$     ▹ tumour catalogue matrix

**Step 1: Data Bootstrapping**

Obtain the augmented count matrix $C_{\text{aug}}$

bootstrapping each genome *t* times from M(N,p)

**Step 2: Training**

**for** *k* in 1..*K* signatures **do**

    **for** *i* in 1..*n* iterations **do**

      MUSE-XAE.train($C_{\mathrm{aug}}$)

      $C_{\mathrm{pred}}$=MUSE-XAE.predict(*C*)

      Collects $E_{\mathrm{rec}}=||C-C_{\mathrm{pred}}|{|}_{F}$

      Collects $W_{ik}$

**Step 3: Clustering**

**for** each *k* in 1..*K* **do**

   K-Means clustering with matching on $\left\{W_{1k},\ldots ,W_{nk}\right\}$

   Obtain consensus Signatures matrix $S_{k}$

**Step 4: Filtering**

Given threshold $th_{\text{avg}}$ and $th_{\text{min}}$

Filter solutions with

$\underset{\text{avg}}{\mathrm{silhouette}}>th_{\text{avg}}$ and $\mathrm{silhouett}e_{\text{min}}>th_{\text{min}}$

**Step 5: Optimal Solution Selection**

Sort filtered solutions based on $E_{\mathrm{rec}}$,

The optimal solution is the one with the lowest $E_{\mathrm{rec}}$

Specifically, given a tumour catalogue matrix $C\in R^{m\times 96}$, where *m* is the number of samples and 96 is the number of mutational channels, for each cancer genome with *N* total number of mutations, we determined the relative mutation frequency *p* for each of the 96 mutational classes. Then, we generated new data points by bootstrapping the cancer genomes *t* times through a multinomial distribution M(N,p), obtaining the augmented count matrix $C_{\mathrm{aug}}$. This approach has already been used by other tools to ensure the stability of a consensus signature (Alexandrov *et al.* 2013a, 2020, Islam *et al.* 2022). Here, we repeated the process *t* times to increase the dataset size. Then, to select the optimal number of active signatures $K^{*}$, we used a revised version of the NMFk approach, originally described by (Nebgen *et al.* 2020) and also adopted by SigProfilerExtractor. Specifically, for each number k=1,…,K of candidate signatures, MUSE-XAE was trained *n* times with different weights’ initialization to guarantee a better stability. Subsequently, a custom K-Means clustering with matching and based on the cosine similarity distance was performed on the set of the decoder weight matrices {$W_{1k}$…$W_{nk}$} to find the consensus signature matrices {$S_{k}$}. This custom clustering approach exploits the Jonker–Volgenant algorithm (Jonker and Volgenant 1987) to solve the linear assignment problem (i.e. the matching) and to find k clusters of equal size *n*. Once obtained the set of clusters, whose centroids represent the signature matrices {$S_{k},k=1\ldots K$}, we considered only the solutions with an average and a minimum silhouette scores above a fixed threshold, choosing as the best solution $K^{*}$, which allows the minimum reconstruction error of the original matrix of cancer samples.

A detailed description is available in Supplementary Section S1 and Supplementary Fig. S1. The size factor *t* for data augmentation, the number of repetitions *n* for each candidate signature *k* and the thresholds for the average and the minimum silhouette scores, i.e. $th_{\text{avg}}$ and $th_{\text{min}}$, can be specified by the user. The open-source code is available at https://github.com/compbiomed-unito/MUSE-XAE.

### 2.3 Signatures assignment

Once the profiles of active signatures within a set of genomes have been identified, it is necessary to understand which signatures cause mutations in a genome and in what amount, that is, we need to assign the contribution of each extracted signature to each genome. Therefore, we used a slightly modified version of MUSE-XAE for the signature extraction. Specifically, we normalized the computed consensus matrix $S_{k}$ into $S_{k_{\mathrm{norm}}}$, which is used to initialize the weights of the decoder, and then freeze them, so that the decoder is no longer trainable and only the weights of the encoder are trained.

To obtain a sparse representation and to avoid over-assignments of the mutational signatures, we used an L1 penalty both for the weights of the last layer of the encoder and for the output of the encoder after a ReLU activation, training the network until convergence. Our new latent representation *z* represents the exposure of the signatures within the genomes, i.e. the number of mutations of a certain mutational class that a signature causes within a genome. We summarized the signature assignment procedure in the Algorithm 2.

Algorithm 2. Signature assignment procedure

Given $C\in R^{m\times 96}$   ▹ SBS catalogue matrix

**Step 1: Signature Profiles Normalization**

Normalize the consensus matrix $S_{k}$ from Algorithm 1.

Get $S_{k_{\mathrm{norm}}}$

**Step 2: Initialization and Freezing of Weights**

Initialize and freeze decoder weights with $S_{k_{\mathrm{norm}}}$

**Step 3: Improve Sparsity**

Add an L1 penalty on the weights of the last encoder layer

Add an L1 penalty on the output after activation

**Step 4: Train the network and obtain Exposures**

MUSE-XAE.train(*C*)

Exposures=MUSE-XAE.z

### 2.4 *De novo* extraction scenarios

To evaluate the performance of MUSE-XAE in the context of mutational signature extraction, we considered five realistic synthetic scenarios (ftp://alexandrovlab-ftp.ucsd.edu/pub/publications/Islam_et_al_SigProfilerExtractor/), available from SigProfilerExtractor (Islam *et al.* 2022). Specifically:

- **Scenario 1:** 1000 synthetic samples, modelling a subset of the pancreatic adenocarcinoma dataset from PCAWG. The 11 ground-truth signatures are based on COSMIC.
- **Scenario 2:** 1000 synthetic tumours from flat, relatively featureless mutational signatures, including a mix of 500 synthetic renal cell carcinomas (high prevalence and mutation load from SBS5 and SBS40) and 500 synthetic ovarian adenocarcinomas (high prevalence and mutation load from SBS3), with 11 COSMIC-based signatures.
- **Scenario 3:** 1000 synthetic tumours from signatures with overlapping and potentially interfering profiles, mostly SBS2, SBS7a, and SBS7b. The mutational load distributions were drawn from bladder transitional cell carcinoma (SBS2) and skin melanoma (SBS7a, SBS7b), with 11 COSMIC-based signatures.
- **Scenario 4:** 1000 synthetic tumours emulating a mix of 500 synthetic renal cell carcinomas (high prevalence and mutation load from SBS5 and SBS40) and 500 synthetic ovarian adenocarcinomas (high prevalence and mutation load from SBS3). In this scenario, only 3 COSMIC-based signatures (SBS3, SBS5, SBS40) are present.
- **Scenario 5:** 2700 synthetic samples with mutational spectra matching those in PCAWG, including 300 spectra from each of nine different cancer types: bladder transitional cell carcinoma, oesophageal adenocarcinoma, breast adenocarcinoma, lung squamous cell carcinoma, renal cell carcinoma, ovarian adenocarcinoma, osteosarcoma, cervical and stomach adenocarcinoma. The ground-truth signatures are 21 signatures based on COSMIC.

We extracted the mutational signatures from each of these scenarios using MUSE-XAE and applied the same performance metrics as in (Islam *et al.* 2022). We used the Hungarian algorithm (Jonker and Volgenant 1987) to match predicted and known signatures according to the cosine similarity. Since the signatures in each scenario are known, an extracted signature was considered correctly identified, or a True Positive (TP), if the cosine similarity between extracted and real signatures was ≥threshold. If the profile of a signature is missing or the cosine similarity was <threshold, it was considered a False Negative (FN) or Positive (FP), respectively.

For each scenario, precision, sensitivity, and *F*1 score were calculated from the corresponding confusion matrices at different cosine similarity thresholds, ranging between 0.8 and 1. A description of the evaluation metrics was reported in Supplementary Section S2.

### 2.5 Real world datasets

To evaluate the performance of MUSE-XAE also in real world scenarios, we applied our method to both the Pan-cancer Analysis of Whole Genomes (PCAWG) dataset, including 2780 tumour samples, and a WGS cohort of 1865 genomes collected from various studies and including the International Cancer Genome Consortium (ICGC) (The ICGC/TCGA Pan-Cancer Analysis of Whole Genomes Consortium 2020), as compiled in Islam *et al.* (2022). For both datasets, we performed: (1) a *de novo* extraction of mutational signatures and a comparison of the profiles with those of SigProfilerExtractor and with the known signatures from COSMIC (Tate *et al.* 2019) and Signal (Degasperi *et al.* 2022) databases; (2) an evaluation of how the signatures and consequently the exposures are discriminative, performing a multiclass classification of the cancer types. Specifically, we used the exposures as new features which were fed into a Random Forest to classify both the primary sites and the cancer subtypes. Finally, we evaluated the performance in terms of balanced accuracy, Matthews Correlation Coefficient (MCC) and Cohen’s Kappa score in a 5-fold cross-validation setting. A description of the metrics is available in Supplementary Section S2.

## 3 Results

### 3.1 Data augmentation improves robustness and accuracy

**Figure 2.**
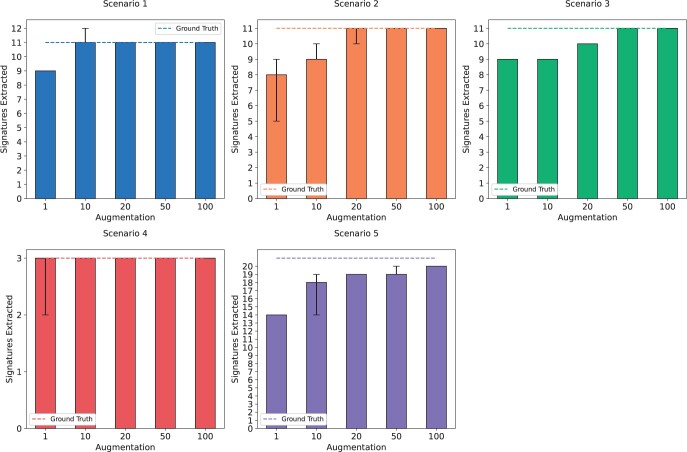
Sensitivity analysis of data augmentation for each of the five synthetic scenario. Each bar represents the average number of extracted signatures over 5 repetitions. The dashed line represents the ground truth, while the error bar represents the range between minimum and maximum.

We first investigated the influence of data augmentation on the extraction of mutational signatures in each of the five synthetic scenarios. Specifically, we performed *de novo* extraction with MUSE-XAE for each of the five datasets, varying the data augmentation level from 1 to 100 times the original dataset size. We repeated the extraction five times at each augmentation level to evaluate stability and accuracy. As depicted in Fig. 2, for the five datasets there is a trend where an increase in data augmentation not only enhances run-to-run stability, but also improves the correct estimation of the real number of profiles.

**Figure 3.**
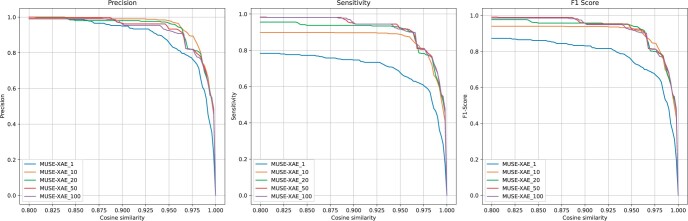
Average precision, sensitivity and *F*1 scores in the five scenarios considering MUSE-XAE at different levels of data augmentation and at varying thresholds of the cosine similarity, ranging between 0.8 and 1.

To further assess the effects of data augmentation, we computed the average precision, sensitivity and *F*1 scores across the five scenarios at different thresholds of the cosine similarity between the extracted and the real profiles, ranging between 0.8 and 1. Figure 3 shows the overall performance. Notably, the sensitivity in the signature profile detection improves with the size of the data augmentation. This confirms that the use of data augmentation is a strategy that improves the detection of signature profiles, and it can be used as an effective technique to further enhance the extraction performance.

### *3.2 De novo* extraction comparison in synthetic scenarios

**Figure 4.**
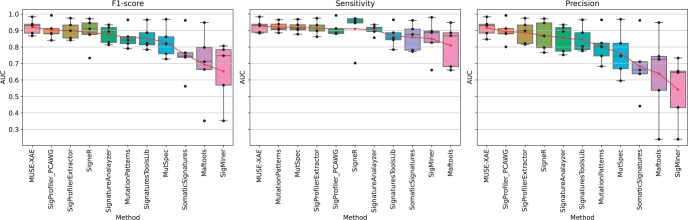
Normalized AUC distribution for *F*1-score, Sensitivity and Precision across the five scenarios for all the tested methods. The methods are ordered according to the average (red dots).

We compared MUSE-XAE (using 100 data augmentation) with 10 state-of-the-art *de novo* signature extraction tools, considering the results reported in ftp://alexandrovlab-ftp.ucsd.edu/pub/publications/Islam_et_al_SigProfilerExtractor/. Precision, sensitivity and *F*1 score were computed in each scenario at different thresholds of the cosine similarity between the extracted and the real profiles, ranging between 0.8 and 1 for each method. Supplementary Fig. S2 shows precision, sensitivity and *F*1 score of the top 10 performing methods averaged across the five scenarios, while Fig. 4 and Supplementary Table S1 show the distribution of the normalized Area Under the Curve (AUC) for the performance scores. In addition, Supplementary Fig. S3 reports, for each scenario, the *F*1 scores at different thresholds of the cosine similarity. Observed results reveal that MUSE-XAE is, on average, the best performing method in all metrics, followed by SigProfilerExtractor and SigProfilerPCAWG.

### *3.3 De novo* extraction in real world datasets

We applied MUSE-XAE for *de novo* extraction of mutational signatures in 2780 samples from PCAWG (18 cancer primary sites and 37 cancer subtypes), and in 1865 samples from an additional extended WGS cohort (15 cancer primary sites and 23 cancer subtypes). We used MUSE-XAE with the data augmentation strategy (i.e. 100 times the original dataset size for 100 iterations) to find stable consensus signatures. MUSE-XAE found 22 and 23 mutational signature profiles in the PCAWG and the extended WGS cohort, respectively. Their profiles are presented in Supplementary Figs S4 and S5.

**Figure 5.**
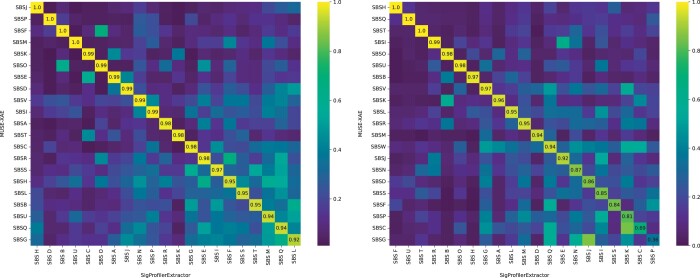
Cosine similarity heatmap between the most similar signatures extracted by MUSE-XAE and SigProfilerExtractor for PCAWG (left panel) and WGS-extended (right panel) cohorts.

By matching the 22 profiles identified by MUSE-XAE with the 21 found by SigProfilerExtractor in the PCAWG cohort, the two methods extracted 21 highly similar profiles, showing a mean cosine similarity of 0.98, with a minimum of 0.92. (Fig. 5, left panel). On the other hand, in the extended WGS cohort, MUSE-XAE found 23 signatures, while SigProfilerExtractor 21, with a mean cosine similarity of 0.90 but a minimum of 0.36 between the 21 most similar signatures. In Fig. 5, it is possible to observe that, in the extended WGS cohort (right panel), although there are 19 out of 21 profiles with a cosine similarity greater than 0.8, the distribution along the diagonal is lower than that one observed in PCAWG (left panel). Moreover, there are two pairs of signatures with a notably low cosine similarity, specifically 0.69 and 0.36, meaning that the two methodologies extract different signature profiles. Therefore, in general, although the two methods are fairly in agreement, MUSE-XAE seems to identify more and different profiles compared to SigProfilerExtractor.

Given that two random 96-component vectors have a cosine similarity of 0.75, and that 0.80 is commonly used as a threshold to determine if two signatures represent the same profile, we can observe from the Supplementary Table S2 that almost all MUSE-XAE profiles from the PCAWG cohort are in agreement with the known signatures from COSMIC and Signal databases. An exception is MUSE-SBSV, which shows a cosine similarity of 0.78 in both COSMIC and Signal databases, potentially indicating an incomplete extraction of the original signature. Conversely, in the WGS-extended cohort, despite most extracted profiles align well with those in COSMIC and Signal databases (Supplementary Table S3), there are three signatures (MUSE-SBSP, MUSE-SBSS, and MUSE-SBSW) showing a cosine similarity below 0.75 with the matched signatures in both databases. Therefore, we further investigated the exposures of these three signatures in the WGS-extended cohort. Notably, as shown in the Supplementary Fig. S6, MUSE-SBSW is predominantly observed in Eye-Melanoma samples (32 out of 46), indicating that it could be a tumour-specific signature. To validate this hypothesis, we performed a *de novo* extraction exclusively for Eye-Melanoma samples, which revealed a strikingly similar profile, showing a pairwise cosine similarity of 0.94 with the one extracted from the pan-cancer analysis. Given the limited number of samples, this finding reinforces the need for a comprehensive examination of this profile, focusing on its origin and validation in an external cohort. Such an in-depth investigation, however, exceeds the objectives of our study, and it will be a focus of our future research.

### 3.4 MUSE-XAE enhances tumour classification

**Figure 6.**
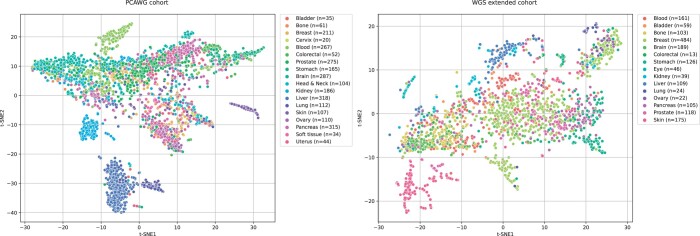
t-SNE representation of the latent representation in PCAWG (left panel) and the WGS-extended (right panel) cohorts, post-hoc coloured by primary tumour sites.

Considering *de novo* extraction of mutational signatures on the real cancer datasets, although COSMIC and Signal databases can be used as reference for the extracted profiles, there is no actual ground truth to calculate the evaluation metrics. Therefore, to thoroughly evaluate the performance of MUSE-XAE, we examined the exposures of the mutational signatures, i.e. the latent representation *z* of tumour samples, both qualitatively and quantitatively. While acknowledging that tumours of the same type may demonstrate a degree of heterogeneity, we assumed that these exposures, representing the mutations caused by a signature within a particular sample, could serve as a key discriminant between different tumour types and subtypes. Figure 6 shows the t-distributed stochastic neighbour embedding (t-SNE) of the latent representations (exposures), coloured by the primary tumour types for both PCAWG (left panel) and the WGS-extended (right panel) cohorts. The t-SNE of exposures displays a clear grouping pattern in both datasets, which provides compelling evidence in support of this hypothesis and indicates a coherent relationship between the signatures exposures and the tumour types.

To quantitatively assess this hypothesis, we implemented a Random Forest Classifier which considers the signature exposures as input features to classify both primary types and tumour subtypes. This classifier was applied to both MUSE-XAE and SigProfilerExtractor exposures using a balanced 5-fold cross-validation approach. To properly train the Random Forest in both datasets, we removed tumour types with less than 10 counts, i.e. the tumour subtypes Myeloid-MDS (*n* = 4), Breast-DCIS (*n* = 4), and Cervix-AdenoCA (*n* = 2) in the PCAWG dataset, while in the WGS-extended dataset we excluded Blood-CMDI (*n* = 9), Sarcoma (*n* = 3), and Bone-cancer (*n* = 2). A complete description of the Random Forest implementation is reported in Supplementary Section S3. Classification performance metrics for primary tumour types and subtypes in PCAWG and the extended WGS cohorts are reported in Tables 1 and 2, respectively.

**Table 1.** Matthews correlation coefficient, Cohen’s Kappa score, and Balanced accuracy metrics calculated by 5-fold cross validation in PCAWG and the extended WGS cohorts for primary tumour type classification.^a^

Model	Dataset	Matthews correlation	Cohen’s Kappa score	Balance accuracy
**MUSE-XAE**	**PCAWG cohort**	0.75(0.02)	0.75(0.02)	0.65(0.02)
SigProfilerExtractor	PCAWG cohort	0.71(0.01)	0.71(0.01)	0.61(0.02)
**MUSE-XAE**	**Extended cohort**	0.73(0.01)	0.72(0.01)	0.65(0.01)
SigProfilerExtractor	Extended cohort	0.70(0.02)	0.69(0.02)	0.61(0.02)

a Metrics are reported as mean and standard deviation. Best performance in bold.

**Table 2.** Matthews correlation coefficient, Cohen’s Kappa score, and Balanced accuracy metrics calculated by 5-fold cross validation in PCAWG and the extended WGS cohorts for tumour subtype classification.^a^

Model	Dataset	Matthews correlation	Cohen’s Kappa score	Balance accuracy
**MUSE-XAE**	**PCAWG cohort**	0.73(0.01)	0.73(0.01)	0.58(0.01)
SigProfilerExtractor	PCAWG cohort	0.67(0.02)	0.67(0.02)	0.52(0.02)
**MUSE-XAE**	**Extended cohort**	0.68(0.03)	0.67(0.03)	0.50(0.03)
SigProfilerExtractor	Extended cohort	0.66(0.02)	0.66(0.02)	0.50(0.03)

a Metrics are reported as mean and standard deviation. Best performance in bold.

In both classification tasks, MUSE-XAE outperformed SigProfilerExtractor across all metrics, suggesting that the exposures and the corresponding signature profiles generated by MUSE-XAE are more discriminative and capable of accurately identifying tumour types. MUSE-XAE particularly outperformed SigProfilerExtractor in the classification of primary tumour types (Table 1), and it discriminates tumour subtypes much better, notably in the PCAWG cohort (Table 2). Supplementary Figs S7–S10 display the confusion matrices of MUSE-XAE for both primary tumour types and subtypes in PCAWG and the extended WGS cohorts. It is worth noticing that MUSE-XAE and SigProfilerExtractor both struggle with the classification of some tumour types. We investigated the possible reason behind considering Breast Cancer as a case study in Supplementary Section S7.

## 4 Discussion

This study introduces MUSE-XAE, a novel method for mutational signature extraction based on an explainable autoencoder. MUSE-XAE combines a nonlinear encoder with a linear decoder by adding a non-negative constraint and a minimum volume regularization. Our method demonstrated high accuracy in the *de novo* extraction of mutational signatures, proven through a sensitivity analysis and a comprehensive comparison with 10 other available tools. In particular, MUSE-XAE resulted as the best performing and the most robust method in different realistic synthetic scenarios, with an average *F*1-AUC of 0.92. In addition, MUSE-XAE identified 22 mutational signature profiles in the PCAWG cohort and 23 mutational signatures in the extended WGS cohort, showing a strong agreement with the known signatures from both COSMIC v3.4 and Signal databases. Notably, in the extended WGS cohort, we found a novel candidate signature specific to Eye-Melanoma. This finding will need to be further investigated and validated in an independent cohort. A detailed investigation of mutational signature exposures revealed that MUSE-XAE profiles are capable of enhancing primary tumour type and subtype classifications. Indeed, the classification performance based on the signature exposures showed MCCs around 0.70 in predicting primary types and tumour subtypes in both PCAWG and the extended WGS cohorts.

MUSE-XAE opens up new possibilities for the development of interpretable neural network-based models for mutational signature extraction, which can leverage the increasing amount of available data and their scalability for larger datasets. Our architecture, given its extreme flexibility, can be used to build more sophisticated models which could integrate the profile of somatic mutations with other clinical and genomic information, potentially improving the extraction of mutational signatures.

## Supplementary Material

btae320_Supplementary_Data

## Data Availability

The data underlying this article are available in the github repository https://github.com/compbiomed-unito/MUSE-XAE.
