# Supplementary material for: MUSE-XAE: MUtational Signature Extraction with eXplainable AutoEncoder enhances tumour types classification
Source: Bioinformatics. 2024 May 16;40(5):btae320. doi: 10.1093/bioinformatics/btae320 (PMC11139523; doi:10.1093/bioinformatics/btae320)
Supplement: btae320_Supplementary_Data [file btae320_supplementary_data.pdf]

MUSE-XAE: MUtational Signature Extraction with eXplainable AutoEncoder enhances tumour types classification

Supplementary Materials

Corrado Pancotti, Cesare Rollo, Francesco Codicè, Giovanni Birolo, Piero Fariselli and Tiziana Sanavia

May 9, 2024

1. MUSE-XAE De Novo Extraction procedure

Given a tumour catalogue matrix $C \in R^{m\times 96}$ with m genomes and 96 mutational classes, the De Novo extraction procedure of MUSE-XAE consists of the following steps:

Data Bootstrapping
For a specific tumour, the augmented count matrix C_{aug} is obtained by bootstrapping each genome t times from a multinomial distribution $M(N, p)$, with N indicating the total number of mutations and p the relative mutation frequency for each of the 96 mutational classes found in that tumour.

Training
For each candidate signature k ($k = 1...K$), MUSE-XAE is trained n times to increase the robustness of the solutions. The training step consists in updating the weights of the autoencoder via a stochastic gradient descent with an Adam optimizer to minimize the composite objective function, consisting in a negative Poisson log likelihood of the augmented C_{aug} matrix with a minimum volume penalty term and a positive constraint on the decoder weights. To avoid overfitting the augmented dataset, an early stopping criterion is implemented. Specifically, at each training epoch, the reconstruction error, E_{rec}, of the original data matrix C is controlled, and the training step is stopped if there is no improvement for a defined number of consecutive epochs. This process is repeated n times for each k signature, obtaining at the end a set of decoder weight matrices $\{W_{1k}, ..., W_{nk}\}$ and a set of reconstruction errors $\{E_{rec_{1k}}, ..., E_{rec_{nk}}\}$ for $k = 1...K$.

Clustering
After the training step, for each $k = 1...K$, a custom K-Means clustering with matching through cosine similarity distance is performed on the decoder weight matrices $\{W_{1k}, ..., W_{nk}\}$, to obtain a consensus matrix S_k of the signatures. Clustering with matching ensures that each group has the same number of points (corresponding to the number of iterations n). Matching means that, for each point, we find the $n - 1$ most similar to it, and this is done using the Jonker-Volgenant algorithm when updating the clusters. Hence, for each k, we finally obtain k clusters whose centroid represents the consensus matrix S_k of the signatures.

Filtering
After clustering, for each $k = 1...K$ the average Silhouette coefficient score and the minimum intra-cluster Silhouette score are calculated. Thresholds th_{avg} and th_{min} are considered candidate solutions if $silhouette_{\text{avg}} > th_{\text{mean}}$ and $silhouette_{\text{min}} > th_{\text{min}}$. Considering both the average and the minimum intra-cluster ensures that the identified signatures are sufficiently separated from each other. The average Silhouette coefficient score is defined as:

$$S = \frac{1}{N}\sum_{i=1}^{N}\frac{b(i) - a(i)}{\max\{a(i), b(i)\}}$$

where N is the total number of points in the dataset, $a(i)$ the average intra-cluster distance for the i-th point, and $b(i)$ the smallest average distance between the i-th point and all the points in any other cluster not including the i-th point.

Optimal Solution Selection Among the set of the candidate solutions satisfying the filtering conditions described above, the best solution is considered as the one with the lowest reconstruction error. Figure S1 illustrates the optimal configuration by plotting the reconstruction error and the average Silhouette score against the number of signatures.

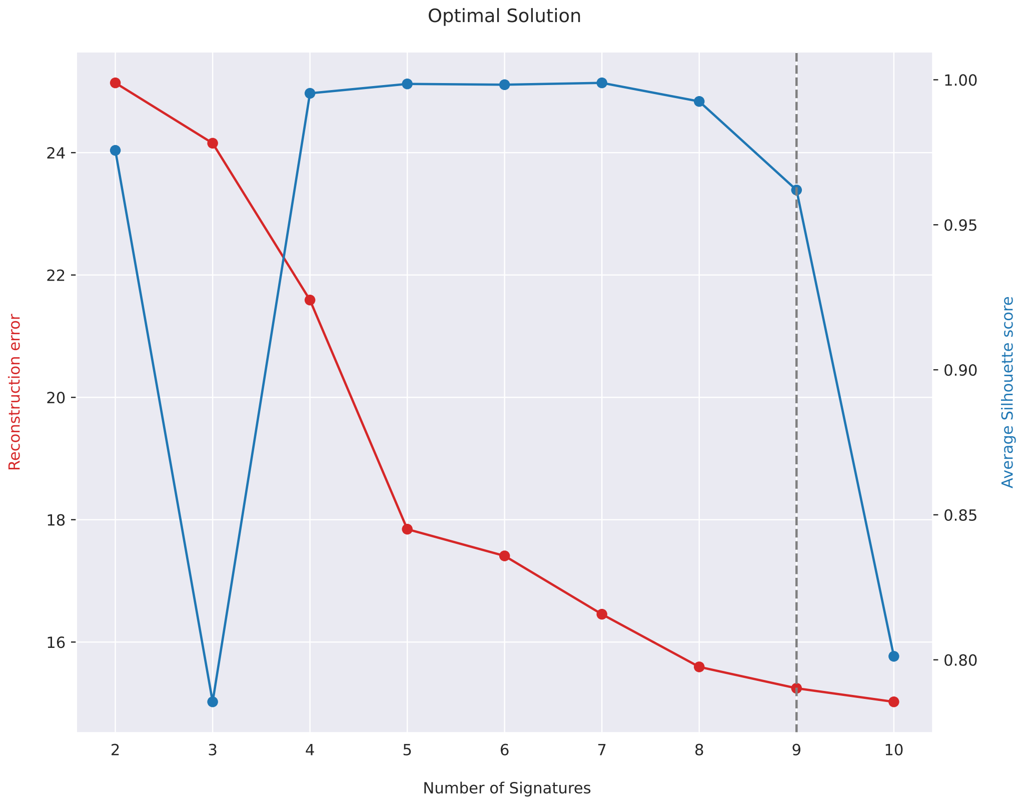

Figure S1: Example of the optimal solution selection plot. On the x-axis the number of mutational signatures, on the y-axis both the reconstruction error (red curve) and the average silhouette score (blue curve). The dashed line represents the optimal solution. In this particular example, the number of mutational signatures is selected according to the thresholds $silhouette_{\text{avg}} > 0.8$ and $silhouette_{\text{min}} > 0.2$

2. Evaluation metrics

2.1 Synthetic scenarios

For each synthetic scenario, we calculated precision, sensitivity, and F1 Score. Each metric is computed by varying the cosine similarity threshold from 0.8 to 1.

$$\textbf{Precision} = \frac{TP}{TP + FP}$$

$$\textbf{Sensitivity} = \frac{TP}{TP + FN}$$

$$\textbf{F1-score} = 2 \cdot \frac{Precision \cdot Sensitivity}{Precision + Sensitivity}$$

Since each metric is considered as a function of the cosine similarity threshold, the Area Under the Curve (AUC) is then computed. Finally, AUC is normalized because the cosine similarity ranges from 0.8 to 1, therefore the maximum AUC value is 0.2 (AUC_{max}):

$$\textbf{AUC}_{norm} = \frac{AUC}{AUC_{max}}$$

This normalization allows an interpretable metric to compare the performance across different methods and scenarios.

2.2 Real World datasets

In PCAWG and WGS-extended cohorts, signatures' exposures are used as features of a Random Forest model to classify both the primary sites and the cancer subtypes. We evaluated the performance in terms of balanced accuracy, Matthews Correlation Coefficient (MCC) and Kohen Kappa score in a 5-fold cross-validation setting. Specifically:

- **Balanced Accuracy**, i.e. the average sensitivity obtained on each class:

$$\text{Balanced Accuracy} = \frac{1}{C}\sum_{c=1}^{C}\frac{TP_c}{TP_c + FN_c}$$

where TP_c and FN_c are the number of true positives and false negatives for the c-th class, and C is the total number of classes.

- **Multi-class Matthews Correlation Coefficient**, defined as:

$$\text{MCC} = \frac{c \times s - \sum_{k=1}^{K}(p_k \times t_k)}{\sqrt{(s^2 - (\sum_{k=1}^{K} p_k)^2) \times (s^2 - (\sum_{k=1}^{K} t_k)^2)}}$$

where $t_k = \sum_{i=1}^{K} C_{ik}$ is the number of times the class k truly occurs, $p_k = \sum_{i=1}^{K} C_{ki}$ is the number of times the class k is predicted, $c = \sum_{k=1}^{K} C_{kk}$ is the total number of samples correctly predicted, and $s = \sum_{i=1}^{K}\sum_{j=1}^{K} C_{ij}$ is the total number of samples.

- **Cohen's Kappa**, which measures the agreement between two raters classifying N items into C mutually exclusive categories each. The formula of the Kappa score is:

$$\kappa = \frac{p_o - p_e}{1 - p_e}$$

where p_o is the relative observed agreement among the raters and p_e is the hypothetical probability of chance agreement.

3. Random Forest Classifier

To test the ability of MUSE-XAE and SigProfilerExtractor in discriminating between different tumour types, a Random Forest classifier was implemented using the exposures of mutational signatures, i.e. the number of mutations generated by a specific signature, as input features. To properly train the classifier, we removed tumour types with less than 10 counts. Specifically, for primary types classification, all tumours have a count greater than 10, whereas for the tumour subtypes we removed Myeloid-MDS (n=4), Breast-DCIS (n=4) and CervixAdenoCA (n=2) in the PCAWG dataset, while in the WGS-extended dataset Blood-CMDI (n=9), Sarcoma (n=3), and Bone-cancer (n=2) were removed. For both methods, the exposures are normalized using min-max scaling and the Random Forest classifier is applied to both datasets using 1000 estimators and considering the Gini criterion to split the trees in the ensemble. Tumour classes are weighted according to their frequency using the "balanced" option in the class weight parameter of the scikit-learn implementation. All the other parameters are set to the default values. Balanced Accuracy, MCC (Matthews Correlation Coefficient), and Kohen Kappa score described above are evaluated in a 5-fold cross-validation setting with same random seed for both methods and both datasets. Cross-validation splits are performed in a stratified manner, according to the frequency of each tumour type.

4. Supplementary Tables

Method	AUC Precision	AUC Sensitivity	AUC F1-score
**MUSE-XAE**	**0.92(0.05)**	**0.93(0.04)**	**0.92(0.05)**
SigProfilerExtractor	0.89(0.07)	0.91(0.04)	0.90(0.06)
SigProfilerPCAWG	0.89(0.07)	0.91(0.05)	0.90(0.06)
SigneR	0.87(0.09)	0.91(0.12)	0.89(0.09)
SignatureAnalayzer	0.85(0.09)	0.90(0.03)	0.88(0.05)
MutationPatterns	0.80(0.11)	0.92(0.03)	0.86(0.07)
SignaturesToolsLib	0.84(0.08)	0.87(0.07)	0.85(0.07)
MutSpec	0.76(0.14)	0.92(0.03)	0.83(0.09)
SomaticSignatures	0.68(0.19)	0.86(0.08)	0.75(0.14)
Maftools	0.64(0.27)	0.81(0.13)	0.69(0.22)
SigMiner	0.54(0.20)	0.85(0.12)	0.65(0.19)

Table S1: AUC_{norm} for precision, sensitivity and F1 score curves for each method, averaged across the five synthetic scenarios. Methods are ordered according to the AUC of the F1 score. Metrics are reported as mean and standard deviation

SBS MUSE-XAE	COSMIC	Cosmic Similarity	SIGNAL	Signal Similarity
SBS A	SBS38	0.95	SBS33	0.92
SBS B	SBS54	0.81	SBS26	0.84
SBS C	SBS12	0.85	SBS12	0.88
SBS D	SBS23	0.93	SBS23	0.92
SBS E	SBS7a	0.97	SBS7a	0.99
SBS F	SBS10a	0.90	SBS10a	1.00
SBS G	SBS44	0.78	SBS44	0.80
SBS H	SBS8	0.88	SBS8	0.94
SBS I	SBS9	0.85	SBS9	0.96
SBS J	SBS1	1.00	SBS96	0.99
SBS K	SBS2	0.99	SBS2	0.99
SBS L	SBS39	0.89	SBS126	0.77
SBS M	SBS22a	0.99	SBS22	1.00
SBS N	SBS43	0.90	SBS99	0.60
SBS O	SBS36	0.97	SBS18	0.89
SBS P	SBS17b	0.93	SBS17	0.99
SBS Q	SBS5	0.65	SBS103	0.86
SBS R	SBS29	0.79	SBS4	0.83
SBS S	SBS92	0.90	SBS16	0.95
SBS T	SBS13	0.90	SBS13	0.99
SBS U	SBS34	0.78	SBS127	0.78
SBS V	SBS40b	0.86	SBS125	0.74

Table S2: Pairwise cosine similarity between matched MUSE-XAE and COSMIC signatures, and between MUSE-XAE and Signal signatures for the PCAWG cohort

SBS MUSE-XAE	COSMIC	Cosmic Similarity	SIGNAL	Signal Similarity
SBS A	SBS9	0.75	SBS9	0.90
SBS B	SBS13	0.92	SBS13	0.99
SBS C	SBS19	0.86	SBS23	0.92
SBS D	SBS26	0.89	SBS123	0.89
SBS E	SBS1	0.85	SBS96	0.86
SBS F	SBS57	0.91	SBS57	0.91
SBS G	SBS12	0.84	SBS16	0.82
SBS H	SBS17b	0.94	SBS17	0.99
SBS I	SBS36	0.91	SBS18	0.86
SBS J	SBS24	0.82	SBS24	0.77
SBS K	SBS7a	0.98	SBS7a	0.99
SBS L	SBS39	0.91	SBS3	0.75
SBS M	SBS6	0.83	SBS1	0.90
SBS N	SBS34	0.84	SBS143	0.76
SBS O	SBS2	0.98	SBS2	0.97
SBS P	SBS32	0.67	SBS32	0.73
SBS Q	SBS43	0.97	SBS99	0.49
SBS R	SBS58	0.97	SBS161	0.57
SBS S	SBS8	0.75	SBS8	0.74
SBS T	SBS22a	0.99	SBS22	0.99
SBS U	SBS38	0.95	SBS38	0.92
SBS V	SBS44	0.96	SBS44	0.88
SBS W	SBS5	0.72	SBS5	0.75

Table S3: Pairwise cosine similarity between matched MUSE-XAE and COSMIC signatures, and between MUSE-XAE and Signal signatures for the WGS extended cohort

5. Supplementary Figures

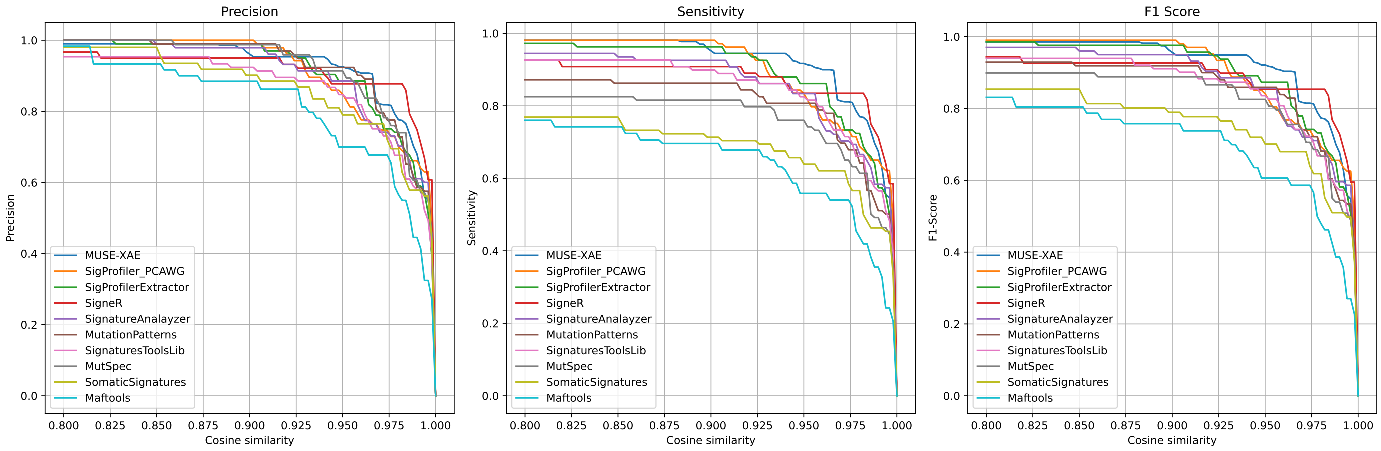

Figure S2: Performance comparison between the top 10 performing methods. On the x-axis the cosine similarity thresholds, while on the y-axis average Precision, Sensitivity and F1 score across the five synthetic scenarios are reported. Methods are ordered according to the F1 score.

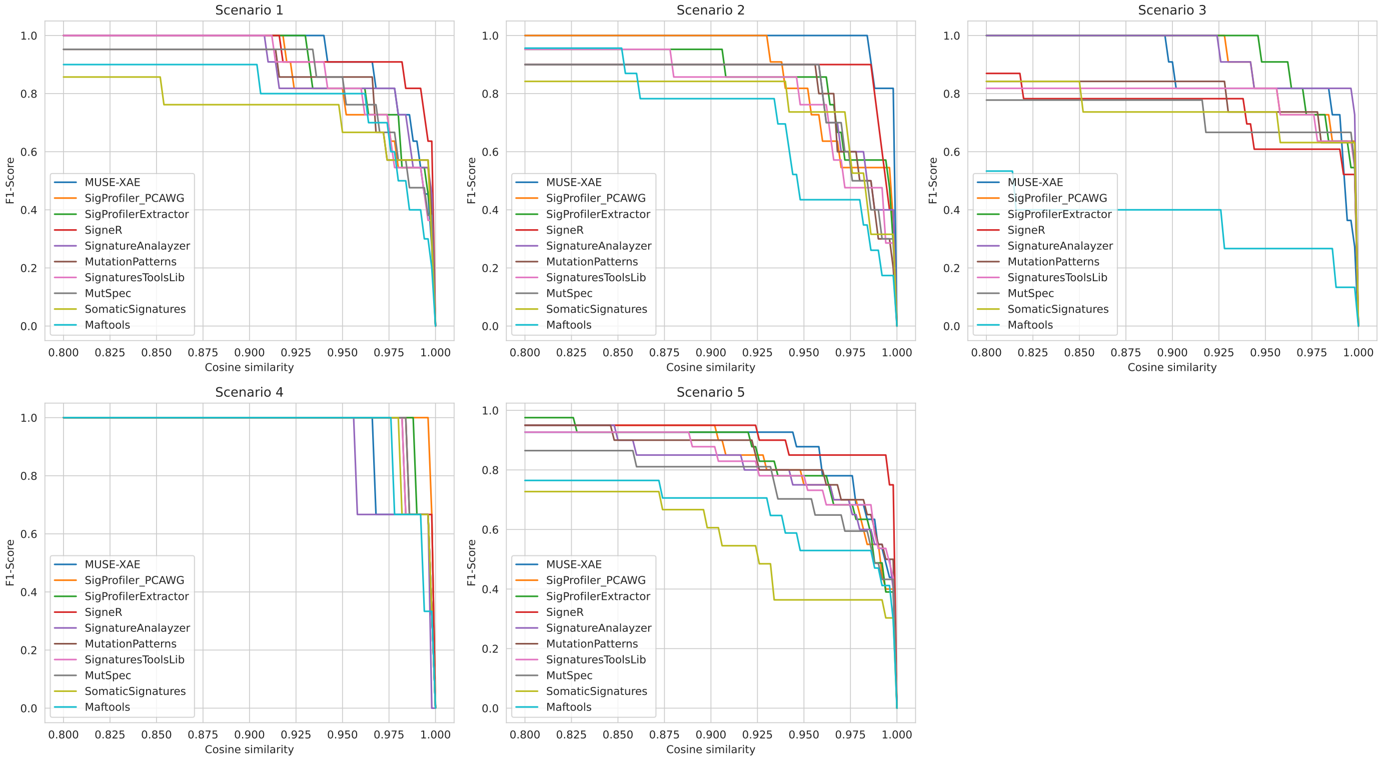

Figure S3: Performance comparison between the top 10 performing methods. On the x-axis the cosine similarity thresholds, while on the y-axis the F1 score for each synthetic scenario are reported. Methods are ordered by the F1 score.

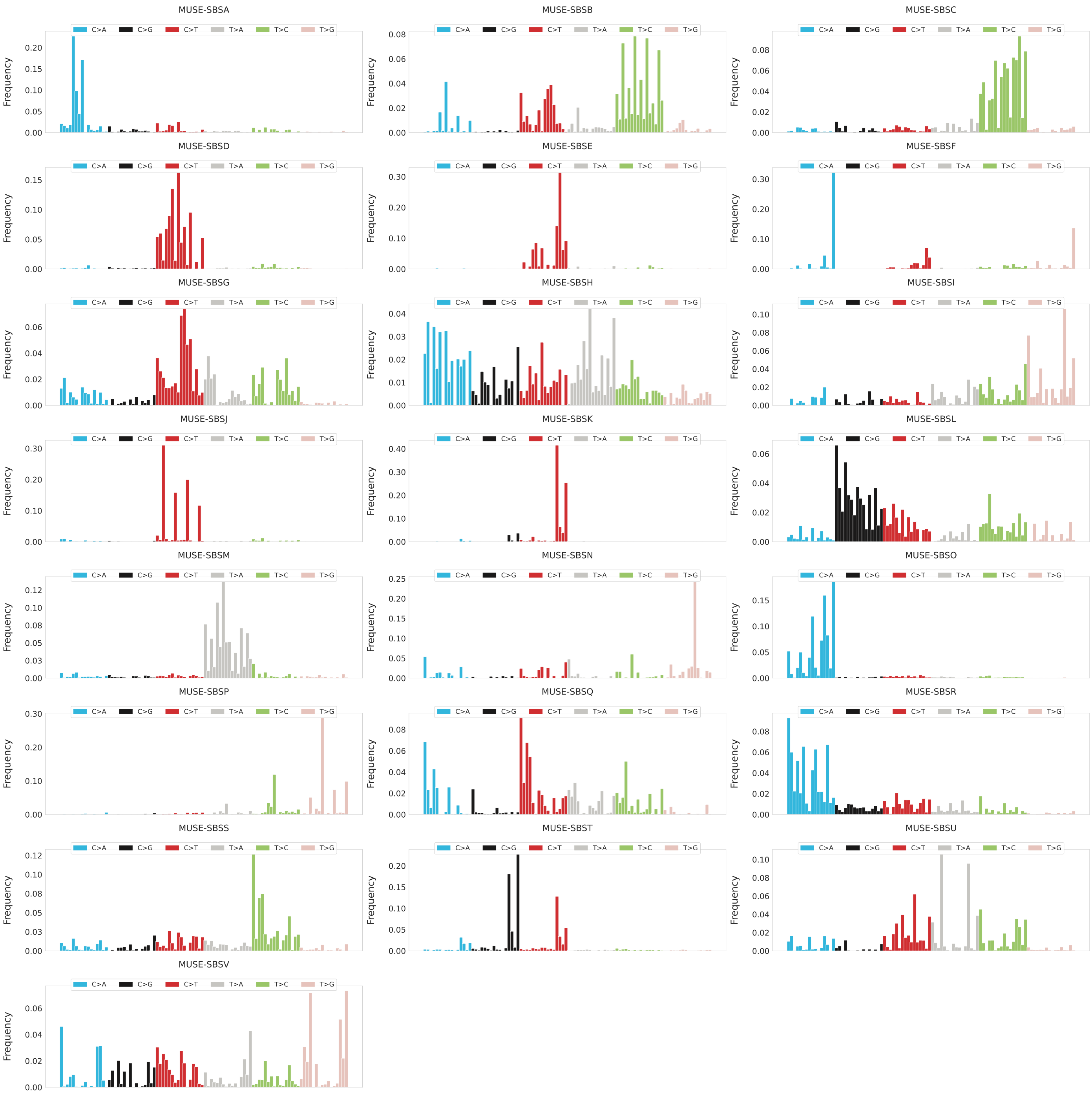

Figure S4: 22 mutational signatures extracted from PCAWG dataset

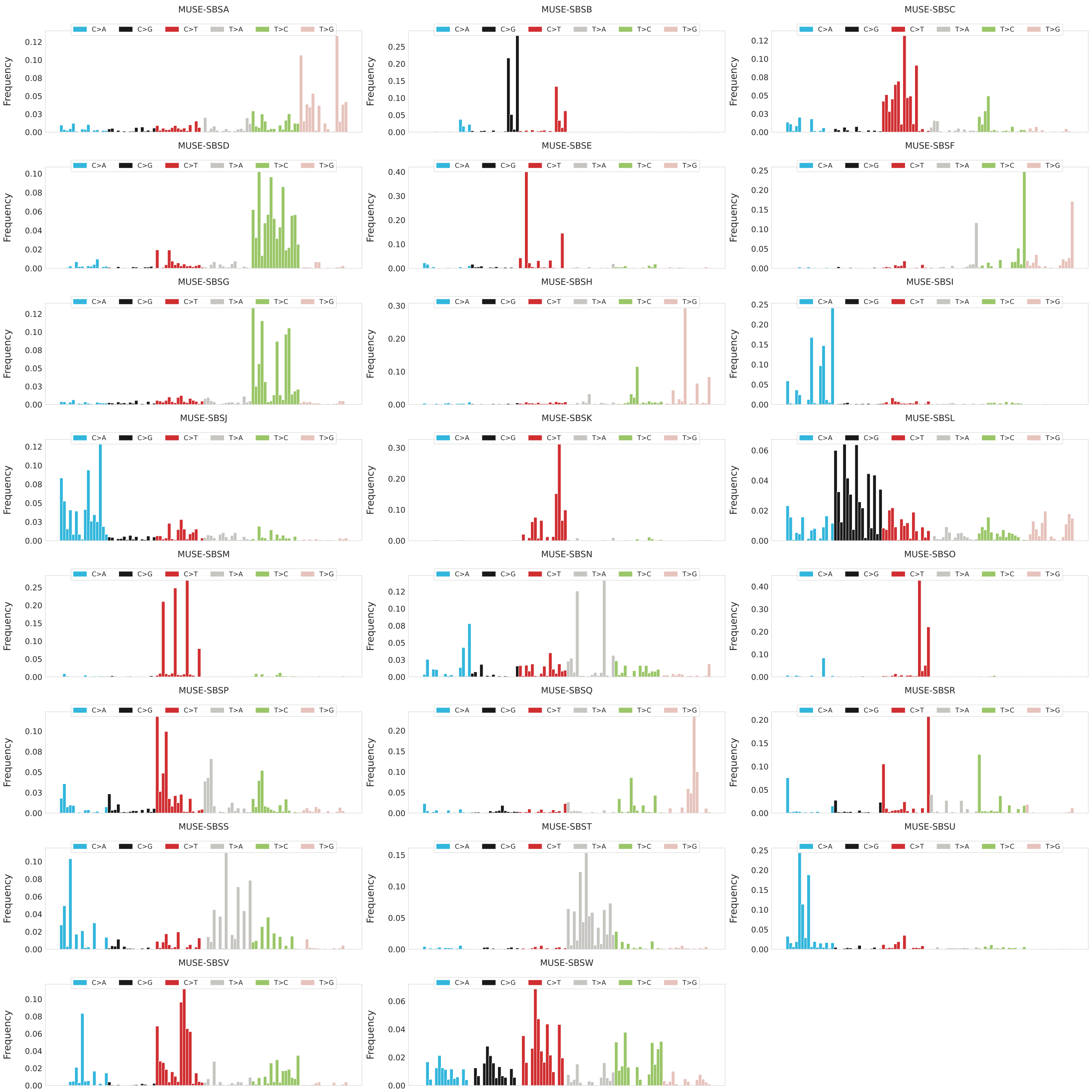

Figure S5: 23 mutational signatures extracted from the WGS-extended cohort dataset

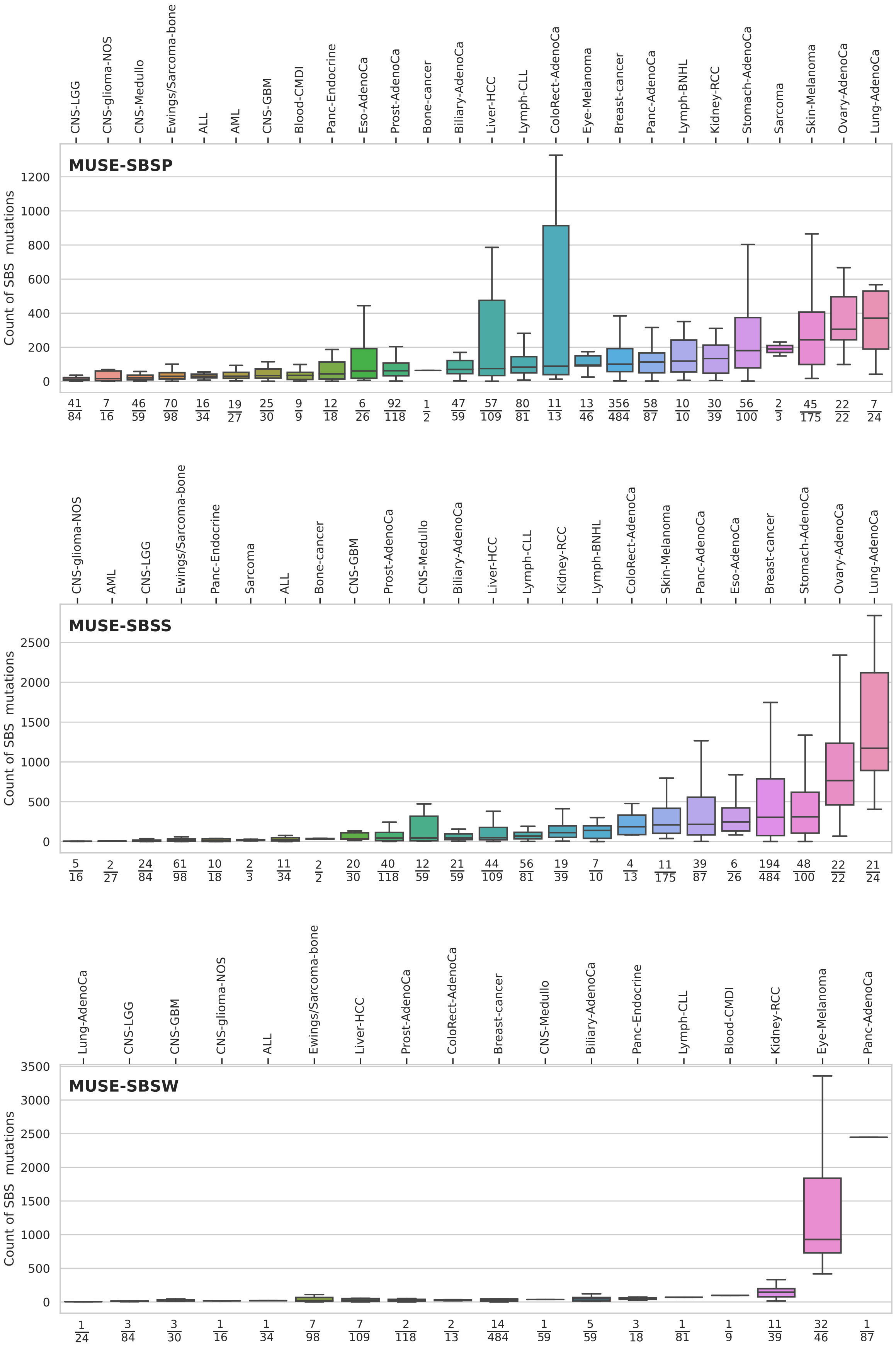

Figure S6: SBS mutation counts for MUSE-SBSP, MUSE-SBSS and MUSE-SBSW for each tumour of the WGS-extended cohort

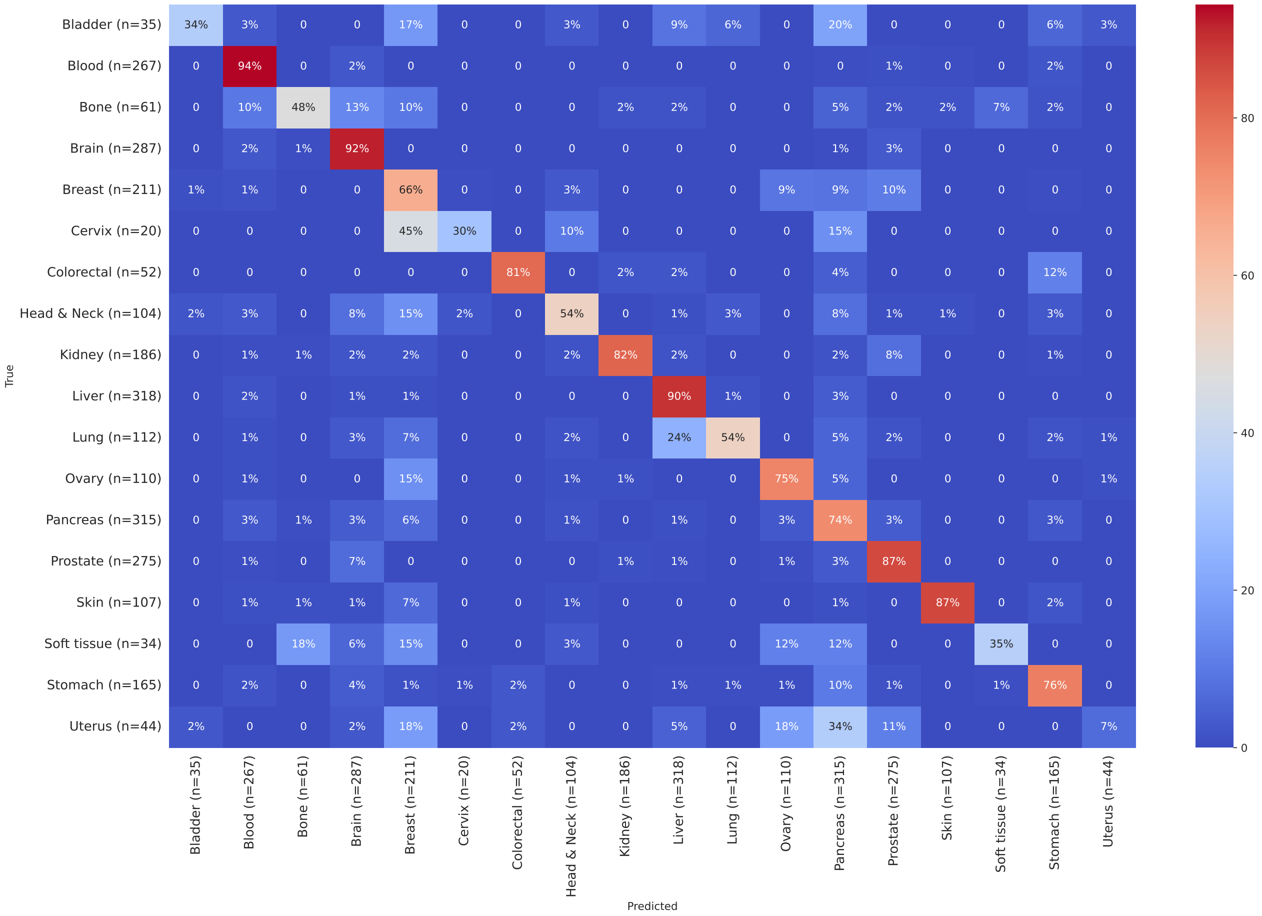

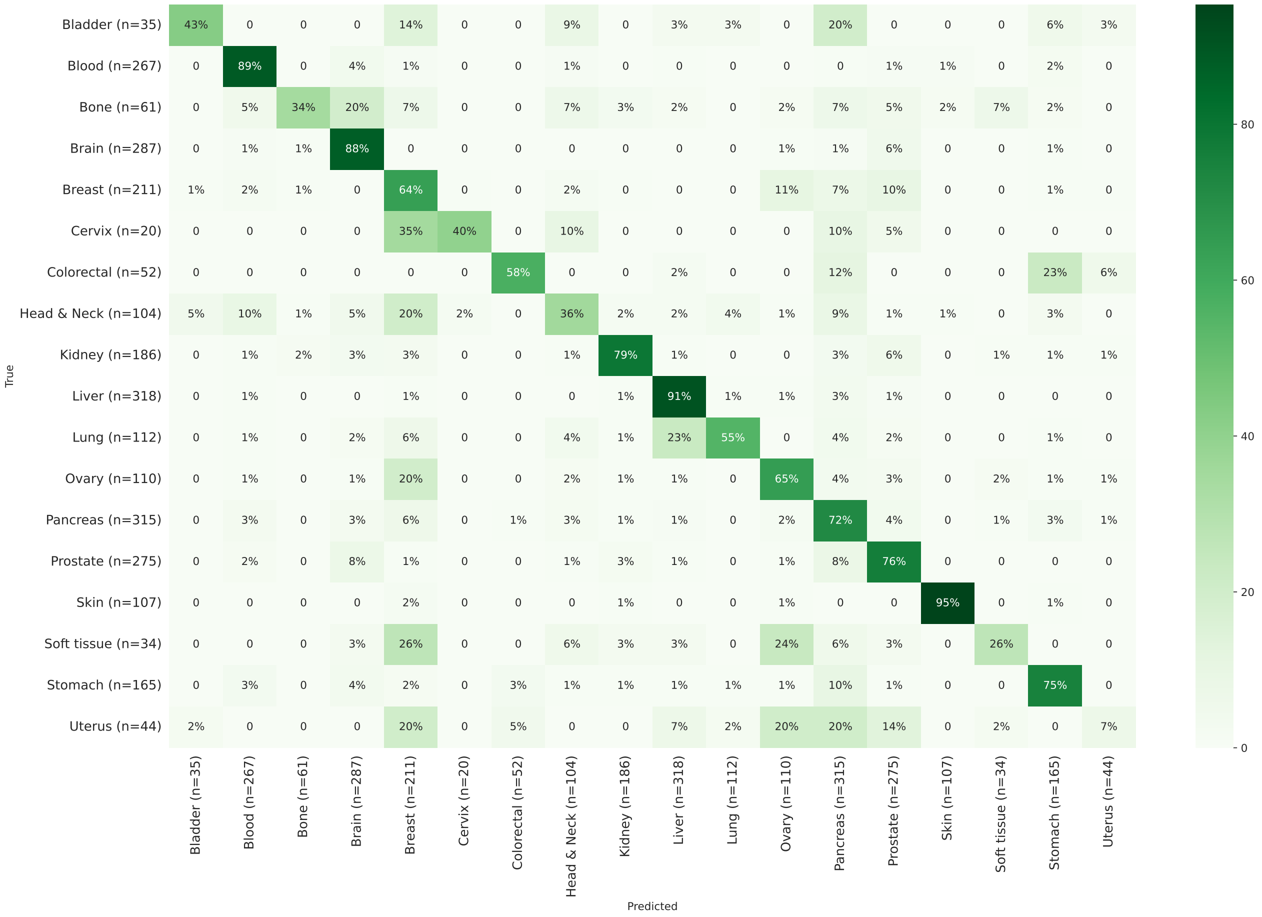

Figure S7: MUSE-XAE (upper panel) and SigProfilerExtractor (lower panel) confusion matrices for 18 PCAWG tumour primary sites

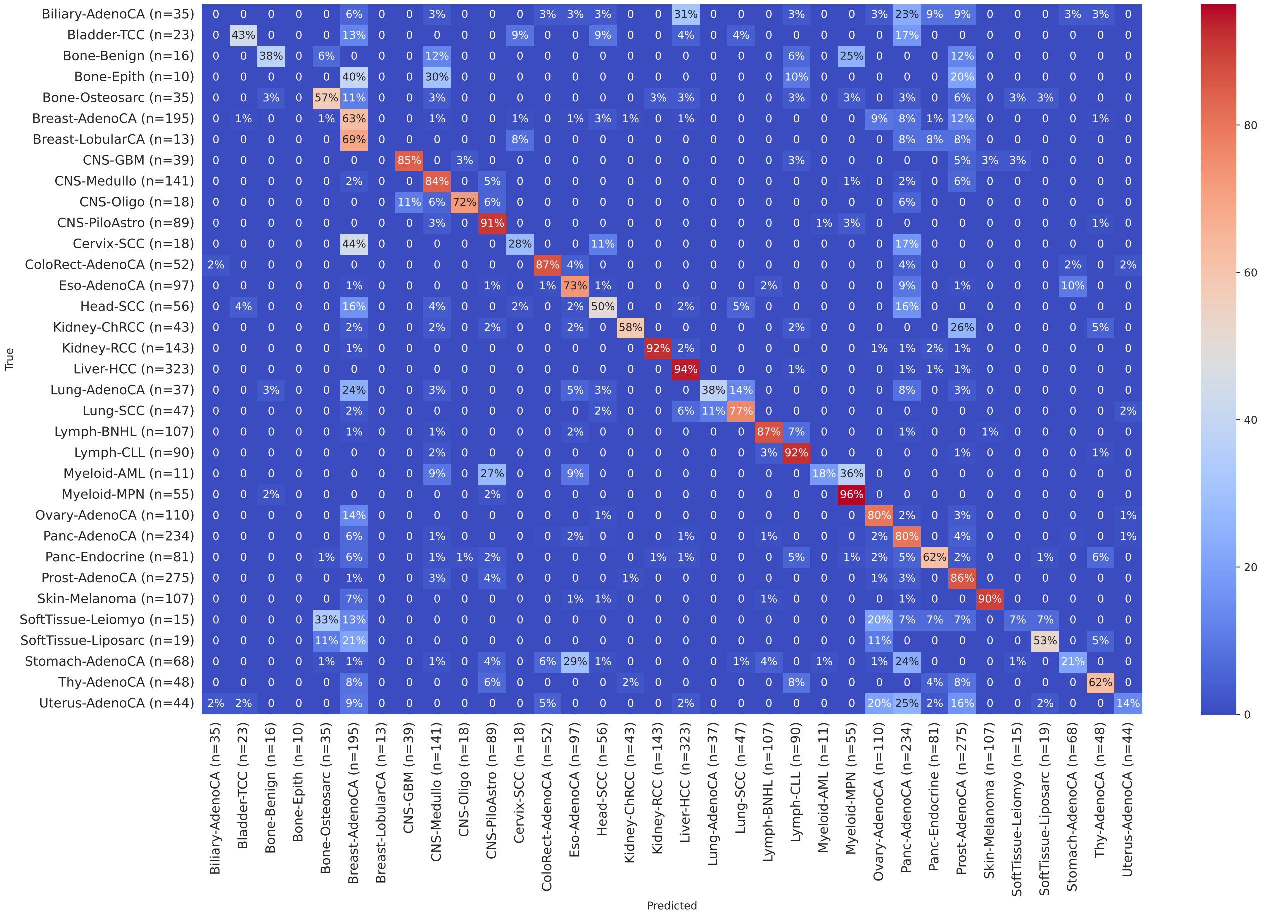

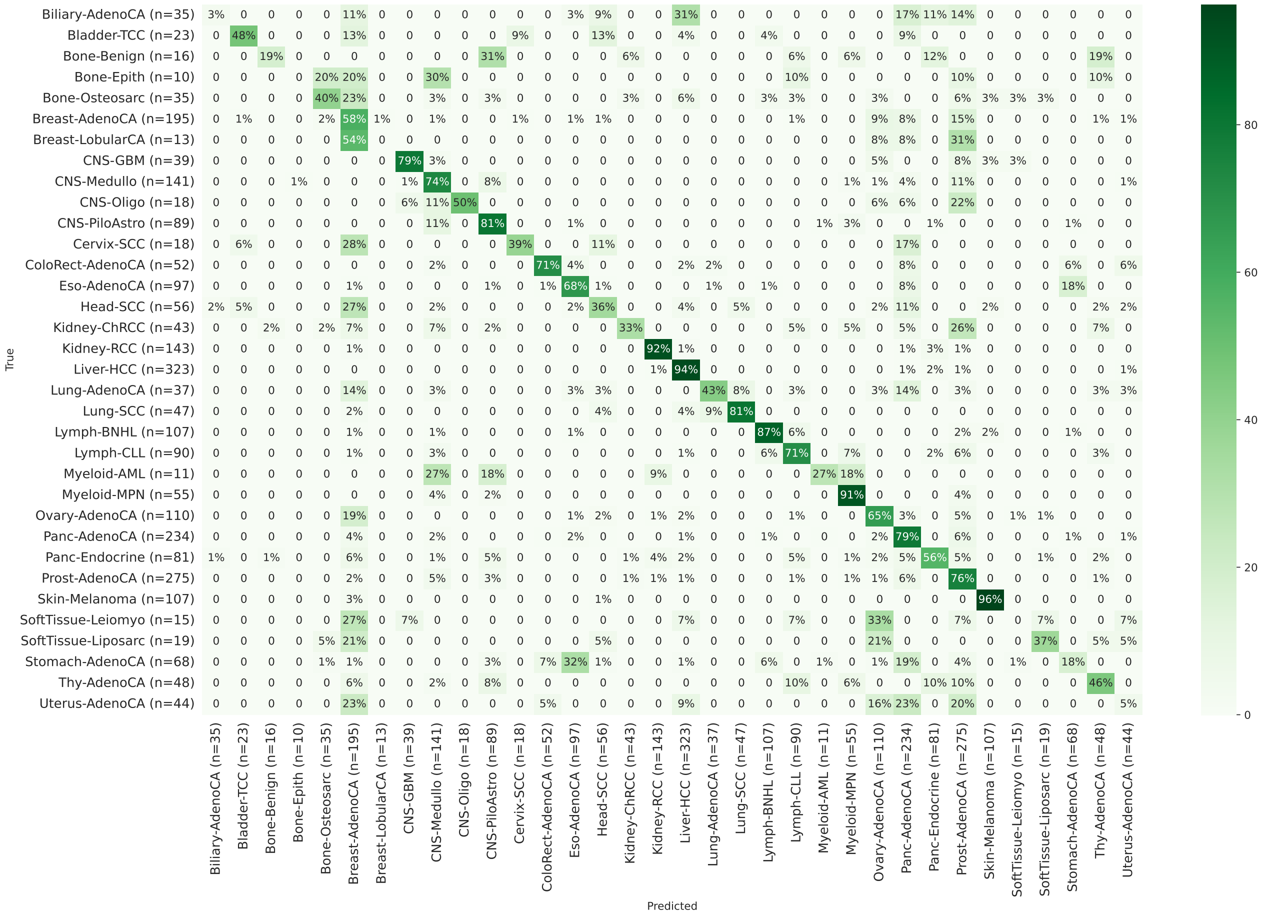

Figure S8: MUSE-XAE (upper panel) and SigProfilerExtractor (lower panel) confusion matrices for 34 PCAWG tumour subtypes.

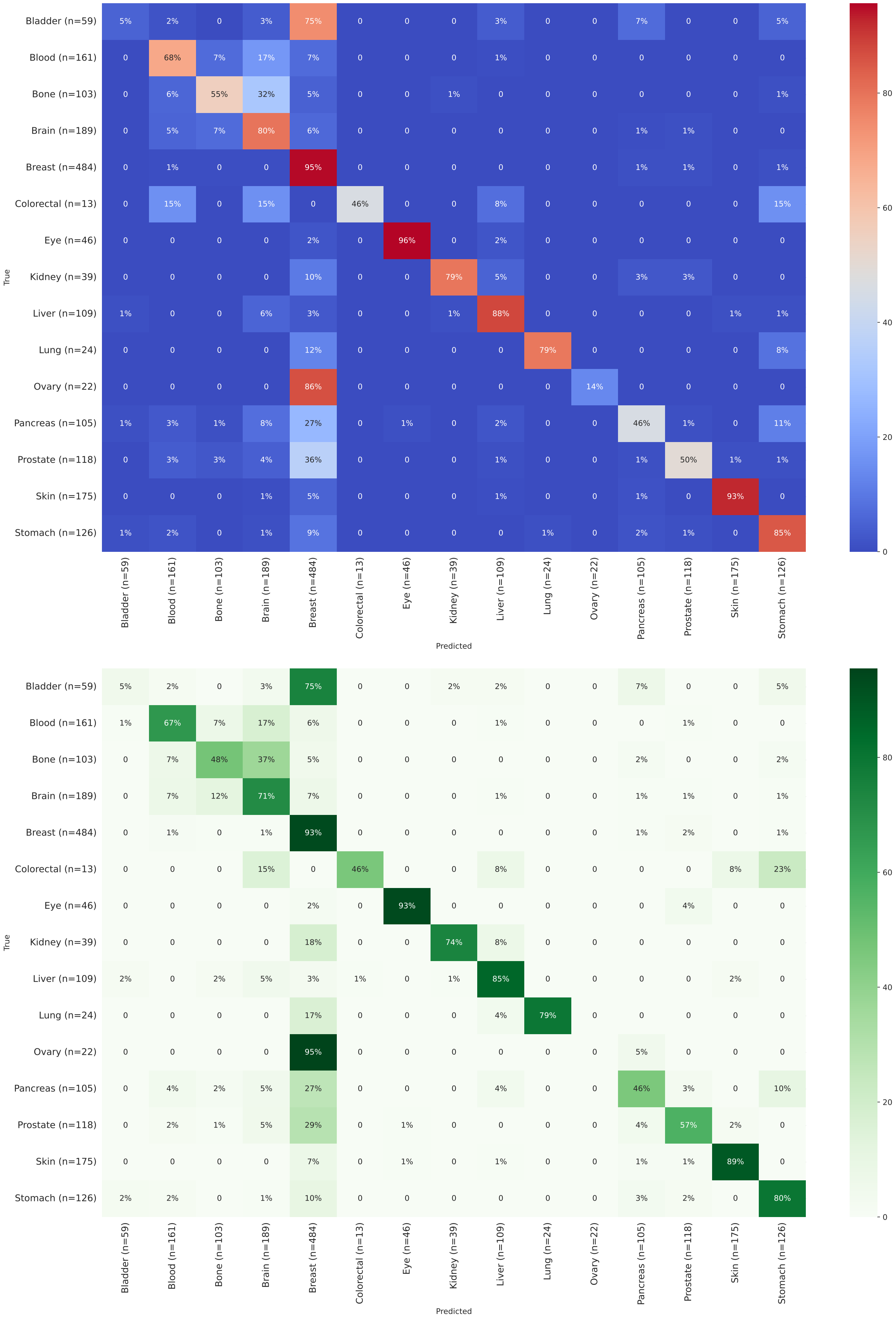

Figure S9: MUSE-XAE (upper panel) and SigProfilerExtractor (lower panel) confusion matrices for 15 WGS-extended tumour primary sites.

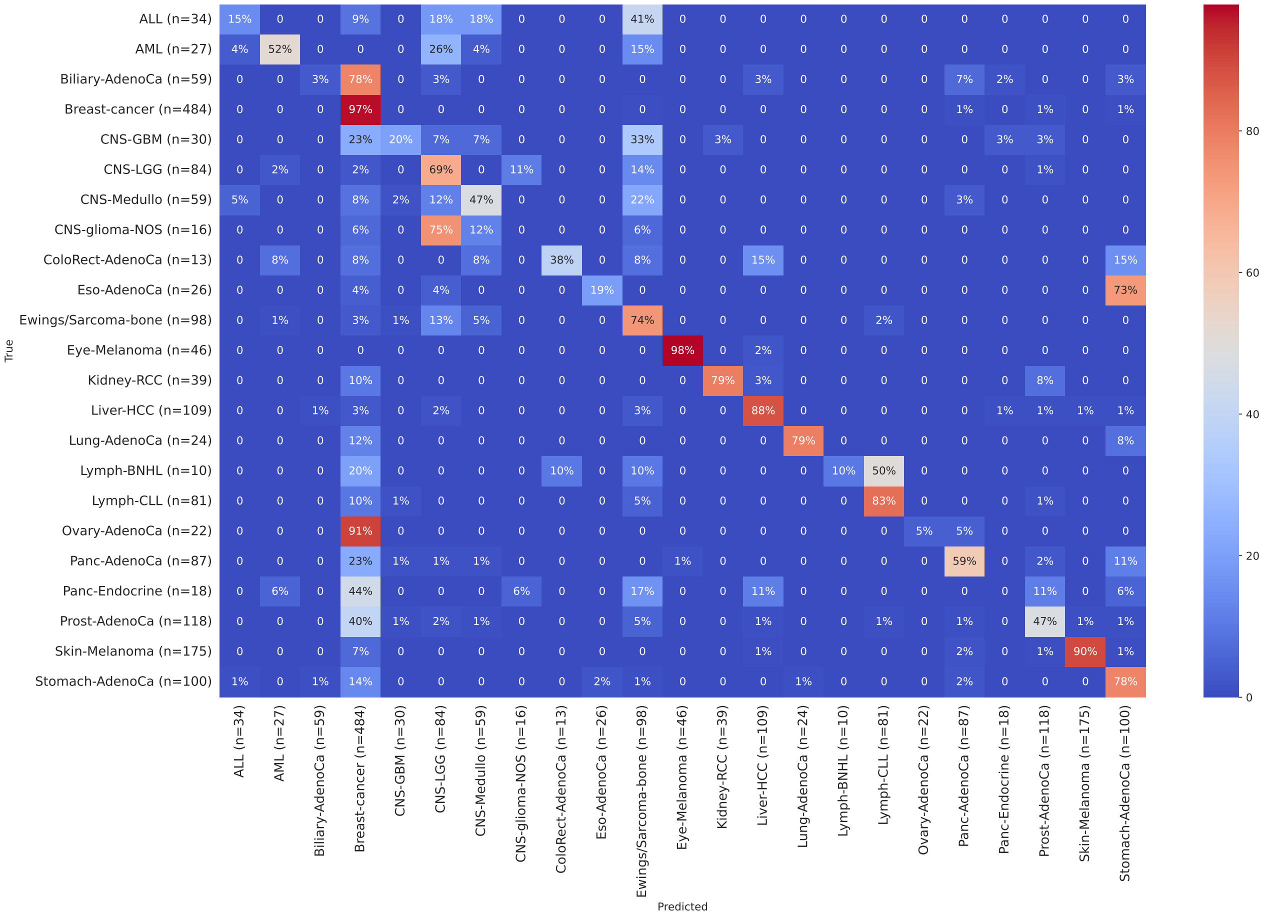

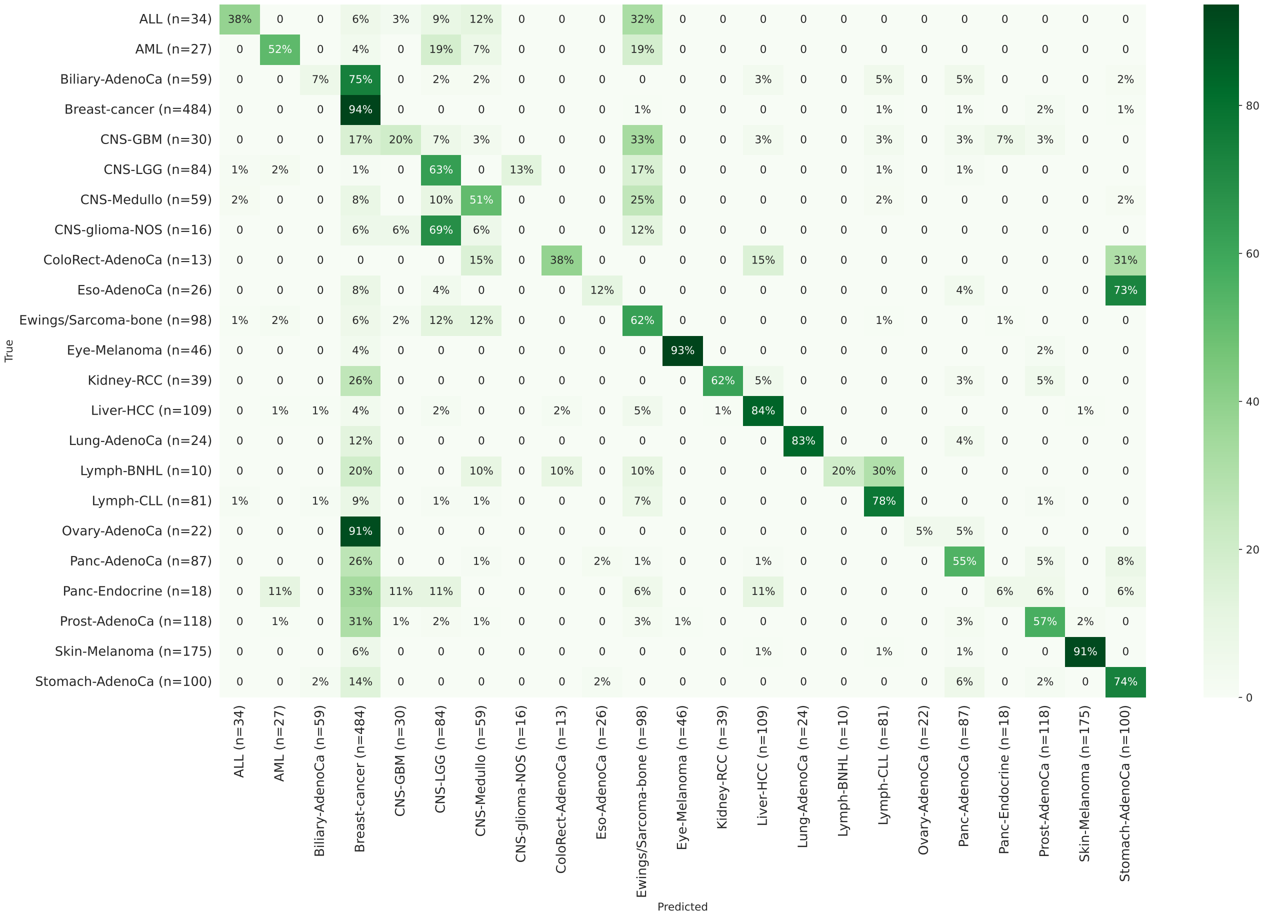

Figure S10: MUSE-XAE (upper panel) and SigProfilerExtractor (lower panel) confusion matrices for 23 WGS-extended tumour subtypes.

6. Signature importance for PCAWG primary types and subtypes classification

We further investigated the key signatures identified as important by the Random Forest model in classifying primary tumor types and subtypes on the PCAWG dataset. Figure S11 displays the shapley-based decision plot of the top 5 most significant signatures for those tumour types that were classified with at least 80% of balanced accuracy (Figure S7). In particular, the decision plot shows for each tumour type how a specific signature contributes to the decision in a one vs. all classification setting, where each line represents a sample. Figure S11 displays the cumulative shapley-based importance on the x-axis: the more positive the value, the more this contributes to classify that tumour as belonging to a specific class. In general, the Random Forest classifier assigns considerable importance to MUSE-SBS J, which has a similarity of 0.99 with COSMIC SBS 1 in a wide range of tumours. SBS 1, prevalent in most cancer types, is associated with a spontaneous mutational process triggered by the deamination of 5-methylcytosine.
MUSE-SBS S, which has a 0.95 similarity with Signal SBS 16, also emerges as significant in numerous tumour types, but with particular importance in liver cancer. This is consistent with previous findings, where SBS 16 was found responsible for many mutations in liver cancer, as reported in the Signal database.
Another significant signature is MUSE-SBS V in kidney samples, which has a similarity of 0.86 with COSMIC SBS 40b. The importance assigned by the Random Forest is in agreement with previous findings for SBS 40b, which was associated with a decreased function of kidney activity, as reported in the COSMIC database.
MUSE-SBS T, which has a similarity of 0.99 with Signal SBS 13, is another signature that is found to be important in various types of tumours, with a greater relevance in brain and blood tumours. SBS13, typically associated with APOBEC activity, is a hallmark signature of several cancers, often found along with SBS2.
Indeed, MUSE-SBS K, showing a similarity of 0.99 with COSMIC SBS 2, is identified as important in multiple cancer types, similar to MUSE-SBS T. These signatures are frequently detected together in the same samples, as noted in the COSMIC database.
Finally, MUSE-SBS E, which has a similarity of 0.99 to COSMIC SBS 7a, is notable as a distinct signature associated with exposure to ultraviolet light and predominantly linked to skin cancer.
Other signatures also contribute to the model, although of less overall importance.

For the above-mentioned tumour types, we also investigated how specific signatures influenced the tumour subtypes classification. Figure S11 shows the decision plot for brain tumours affecting the central nervous system (CNS) and blood tumours (divided into lymphatic and myeloid). Specifically, in addition to the above-mentioned signatures, the decision plot shows the important contribution of MUSE-SBS I in Lymphoid BNHL, which has a similarity of 0.96 with Signal SBS 9, a signature associated with mutations induced during replication by polymerase eta in lymphoid cells, as reported on both Signal and COSMIC databases.
SBS O is similar to COSMIC SBS 36 with 0.97 of similarity and COSMIC SBS 18 with 0.94. Investigation of the COSMIC and Signal databases suggests that it is more plausible that it can be considered the signature SBS 18 since this is in agreement with what was found in CNS and Myeloid tumours.
The same stands for SBS P which has a similarity of 0.93 with COSMIC SBS 17b where precisely this was observed particularly in Lymph-BNHL tumours.

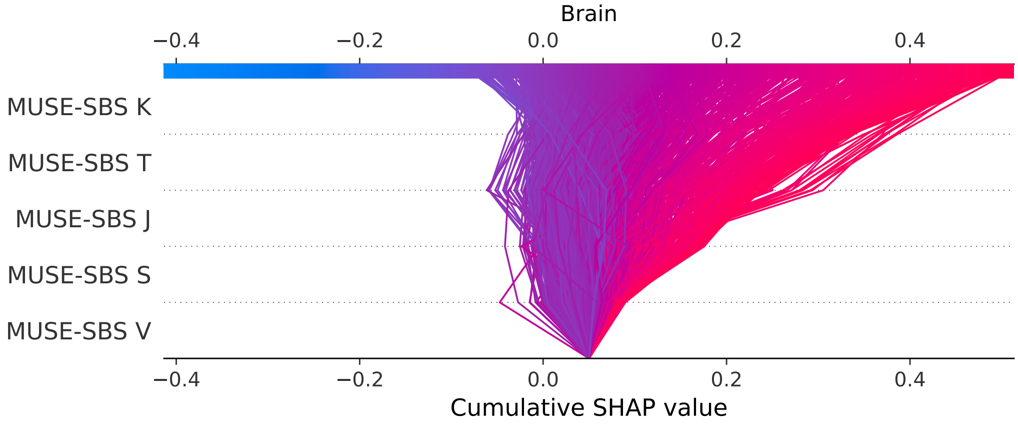

Brain
MUSE-SBS K
MUSE-SBS T
MUSE-SBS J
MUSE-SBS S
MUSE-SBS V
−0.4
−0.2
0.0
0.2
0.4
Cumulative SHAP value

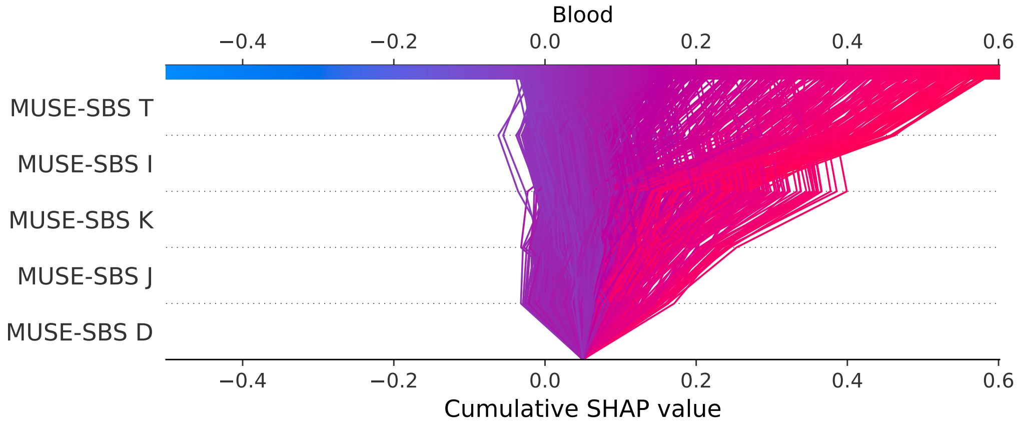

Blood
MUSE-SBS T
MUSE-SBS I
MUSE-SBS K
MUSE-SBS J
MUSE-SBS D
−0.4
−0.2
0.0
0.2
0.4
0.6
Cumulative SHAP value

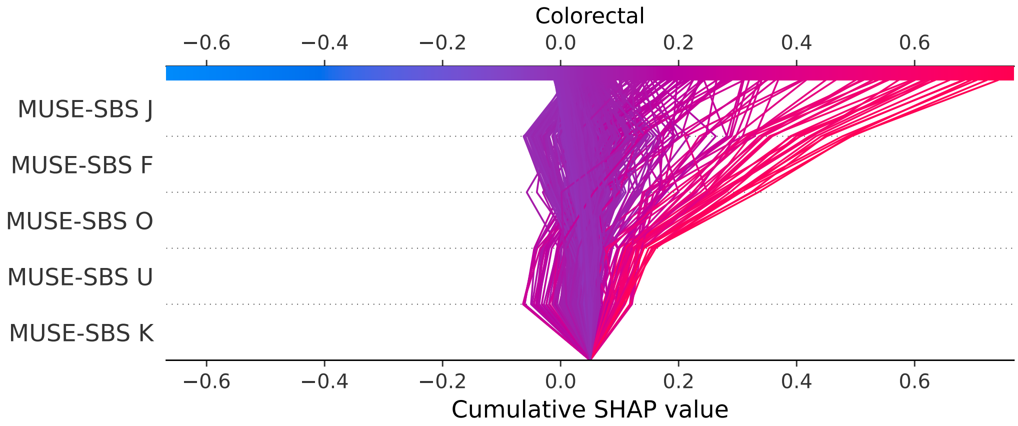

Colorectal
MUSE-SBS J
MUSE-SBS F
MUSE-SBS O
MUSE-SBS U
MUSE-SBS K
−0.6
−0.4
−0.2
0.0
0.2
0.4
0.6
Cumulative SHAP value

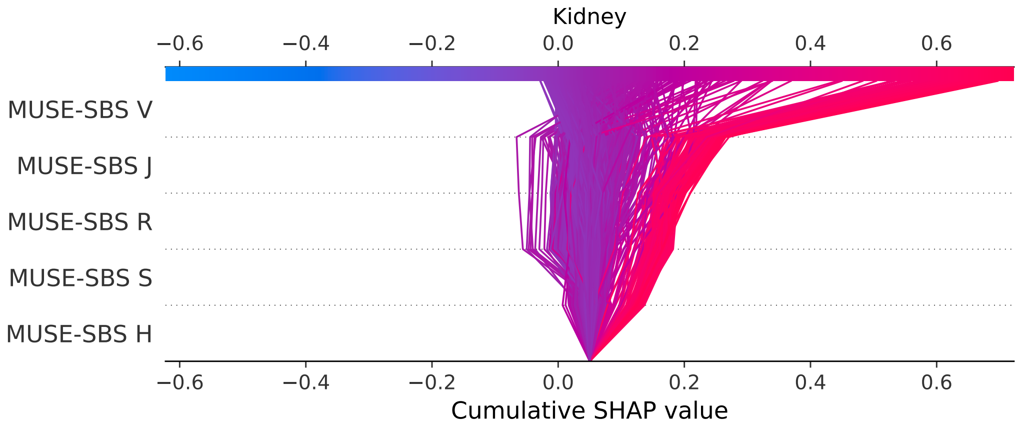

Kidney
MUSE-SBS V
MUSE-SBS J
MUSE-SBS R
MUSE-SBS S
MUSE-SBS H
−0.6
−0.4
−0.2
0.0
0.2
0.4
0.6
Cumulative SHAP value

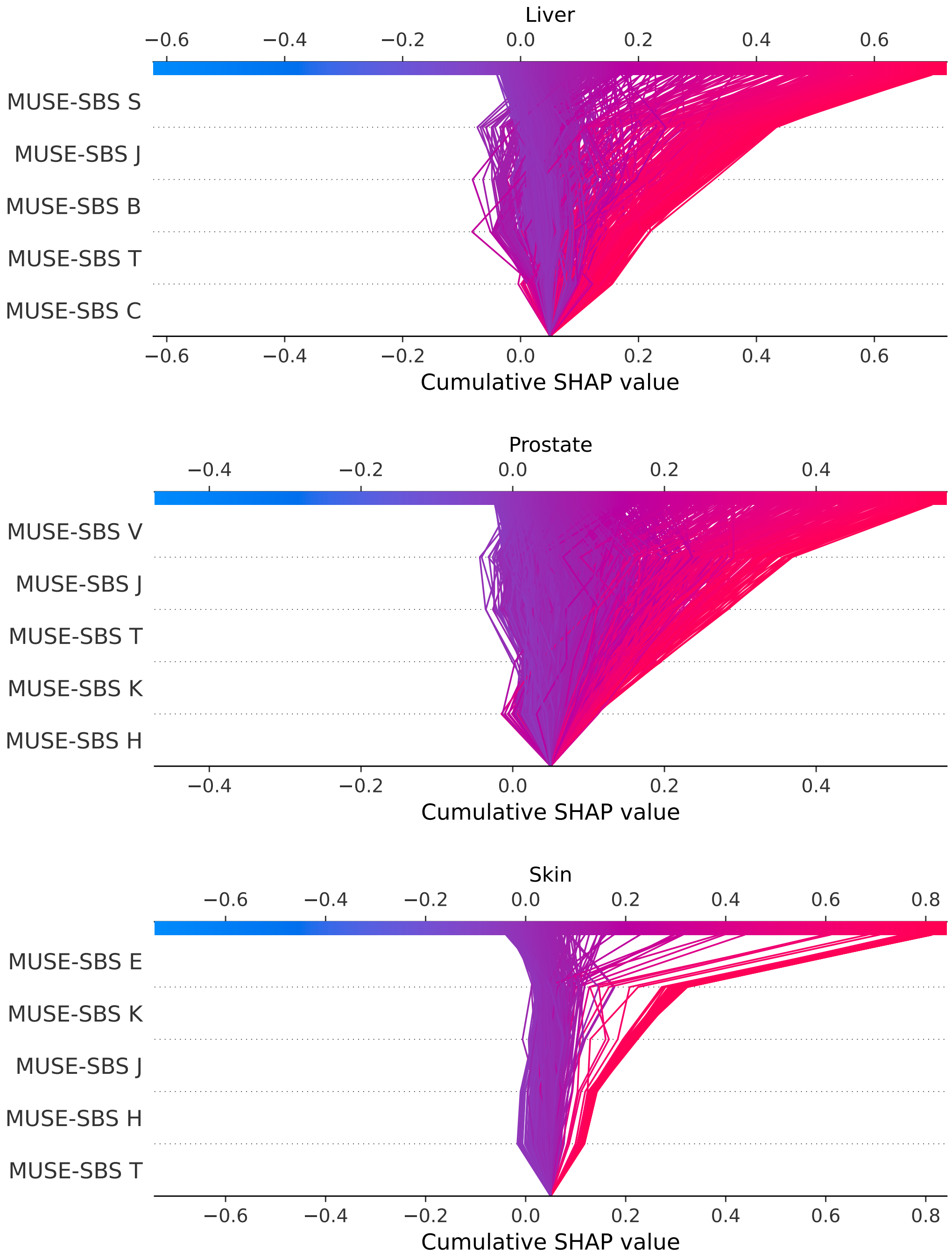

Figure S11: Decision plot for the top 5 features in those PCAWG primary tumours that were classified by the Random Forest with more than 80% of accuracy

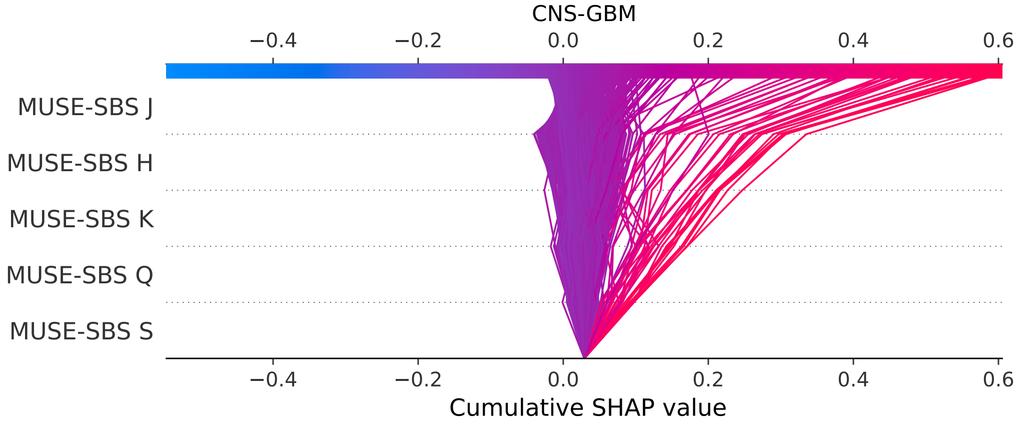
CNS-GBM
MUSE-SBS J
MUSE-SBS H
MUSE-SBS K
MUSE-SBS Q
MUSE-SBS S
−0.4
−0.2
0.0
0.2
0.4
0.6
Cumulative SHAP value

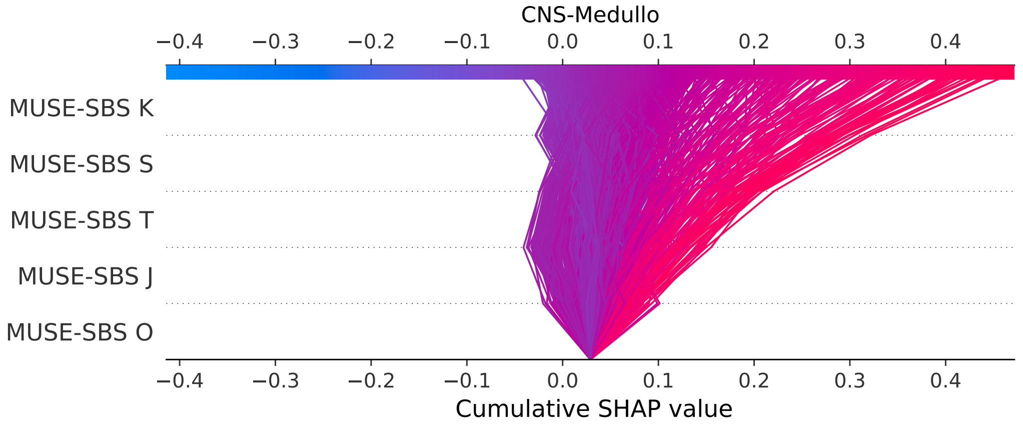
CNS-Medullo
MUSE-SBS K
MUSE-SBS S
MUSE-SBS T
MUSE-SBS J
MUSE-SBS O
−0.4
−0.3
−0.2
−0.1
0.0
0.1
0.2
0.3
0.4
Cumulative SHAP value

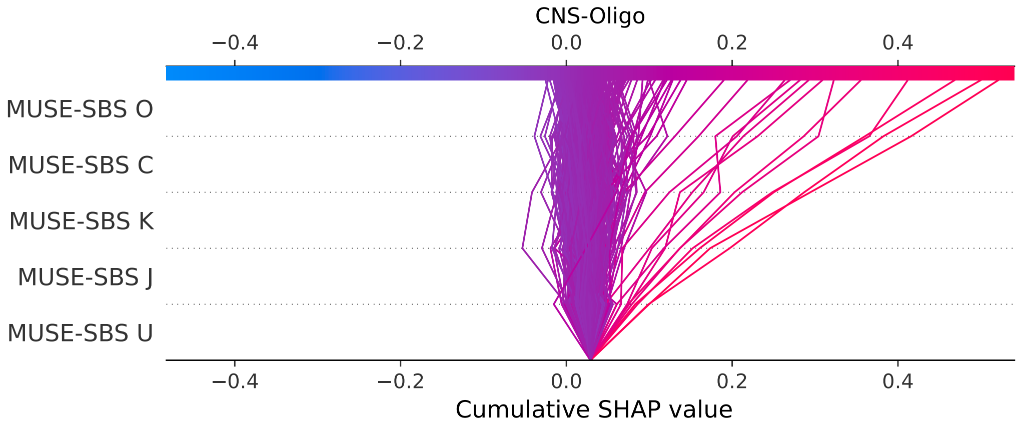
CNS-Oligo
MUSE-SBS O
MUSE-SBS C
MUSE-SBS K
MUSE-SBS J
MUSE-SBS U
−0.4
−0.2
0.0
0.2
0.4
Cumulative SHAP value

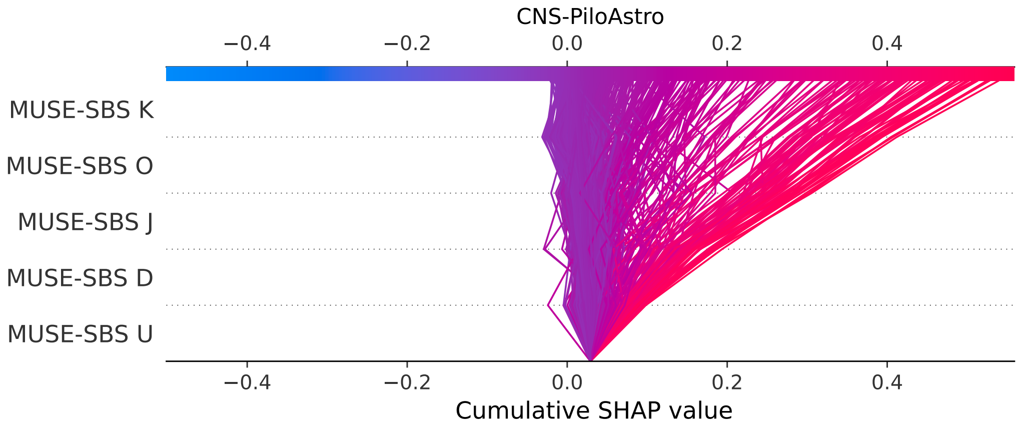
CNS-PiloAstro
MUSE-SBS K
MUSE-SBS O
MUSE-SBS J
MUSE-SBS D
MUSE-SBS U
−0.4
−0.2
0.0
0.2
0.4
Cumulative SHAP value

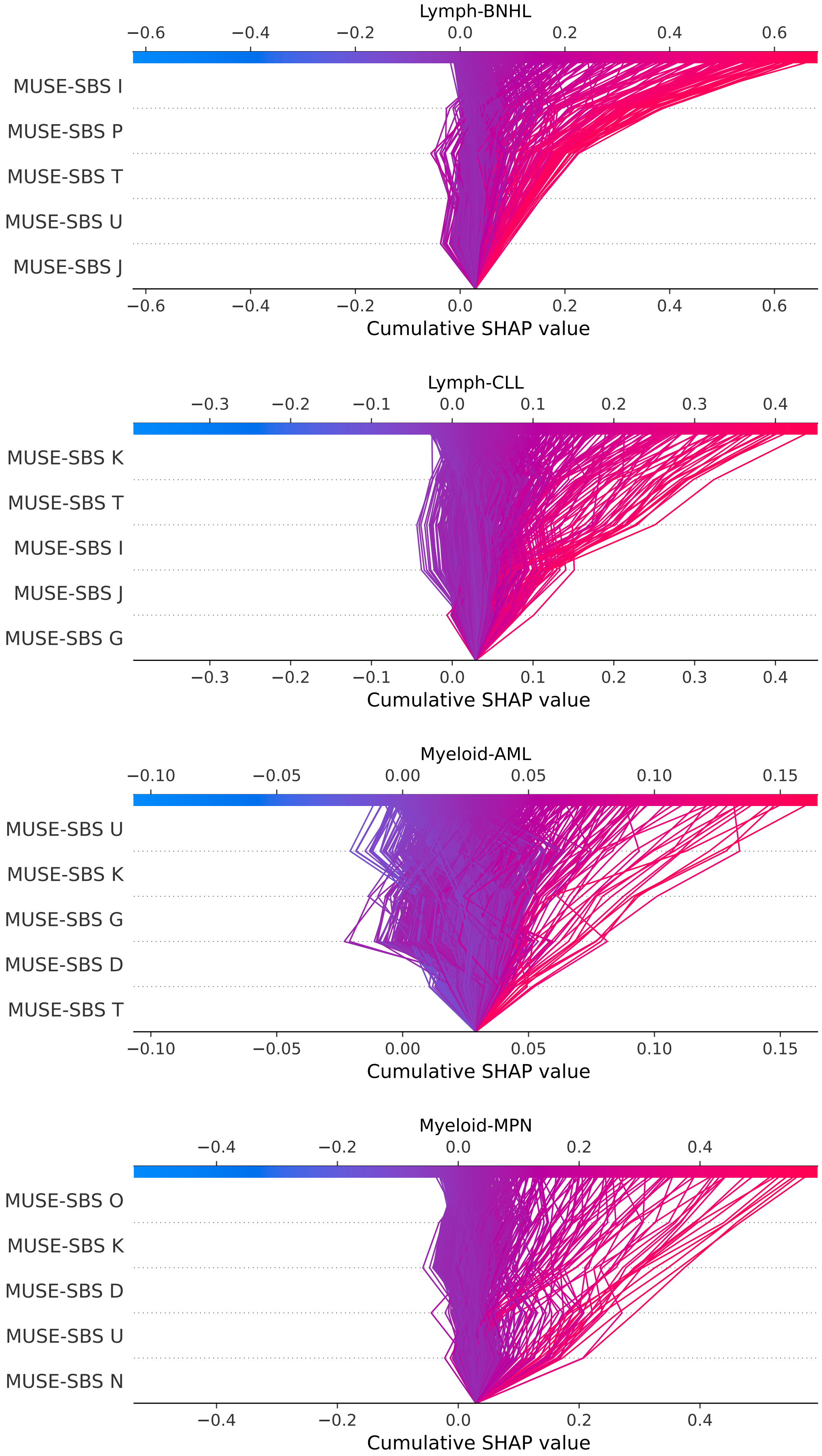

Figure S12: Decision plot for the top 5 features in those PCAWG tumours subtypes that were classified by the Random Forest with more than 80% of accuracy

7. Case study: Breast cancer

From Supplementary Figure S7, it can be observed that some tumours cause the Random Forest classifier to struggle for exposures derived from both MUSE-XAE and SigProfilerExtractor. We have attempted to further investigate the possible reasons, considering Breast cancer as a case study, since the accuracy appears to be much higher in the WGS-extended cohort (95% for MUSE-XAE and 93% for SigProfilerExtractor) than in the PCAWG cohort (66% for MUSE-XAE and 64% for SigProfilerExtractor).
Hence, it seems that the inherent heterogeneity of Breast cancer within the PCAWG dataset challenges the classification task. This complexity likely arises from the heterogeneous molecular profiling characterising Breast cancer subtypes, which cannot be adequately captured through single substitution mutation data alone. Therefore, we first explored the available metadata to identify any discernible differences in the molecular subtypes between the two datasets. Table S4 shows the PCAWG Breast Cancer divided per project with metadata retrieved from [1].

Sample	Characteristics
BRCA-UK (n=44)	Triple negative/lobular
BRCA-EU (n=76)	ER+, HER-
BRCA-US (n=91)	Ductal and lobular

Table S4: Breast Cancer PCAWG cohort

On the other hand, the 484 Breast tumours in the WGS-extended cohort derives from the 560 Breast cancer project [2], which can be stratified by their molecular characteristics, as shown in Table S5:

Sample	Characteristics
n=320	ER+, HER2-
n=46	ER+, HER2+
n=167	Triple negative
n=27	ER-, HER2+

Table S5: Breast Cancer WGS extended cohort

Thus, it appears that all molecular Breast cancer types of the PCAWG dataset are also represented in the WGS-extended cohort, which additionally includes ER-/HER+ and ER+/HER2+ subtypes. However, the Random Forest classifier still classifies almost all Breast tumours in the WGS-extended cohort correctly. Looking at the misclassifications reported by the confusion matrices in the PCAWG cohort (Figure S7), while Breast cancer is correctly classified around 66% of times by MUSE-XAE, about 30% of Breast tumour samples were misclassified as Ovary, Prostate or Pancreatic. This is in agreement with the t-SNE plot of the original mutation data of these tumour types (Supplementary Figure S13, upper panel): even if there are distinct clusters, there are several overlapping points between the tumour types. This is also reflected by the confusion matrices. However, in the WGS-extended cohort the t-SNE plot of the same tumour types shows much more overlapping tumour types (Figure S13, lower panel). Indeed, as shown in Figure S10, in the WGS-extended cohort many samples of Ovary, Prostate and Pancreatic cancers are misclassified as Breast cancer, while, on the other hand, almost all Breast cancer samples are classified correctly, with a high number of false positives. This effect is probably due to the fact that, in general, the mutational profiles along the 96 substitution mutational classes are very similar, but in this particular dataset (WGS-extended cohort) the proportion of Breast samples is much higher than in the PCAWG cohort. This probably leads the Random Forest classifier to favour the Breast cancer class, even if using a balanced version of the classifier. Therefore, given the high similarity of the 96-base mutational profile, it is difficult to accurately discriminate between some tumour types with no other mutational information.

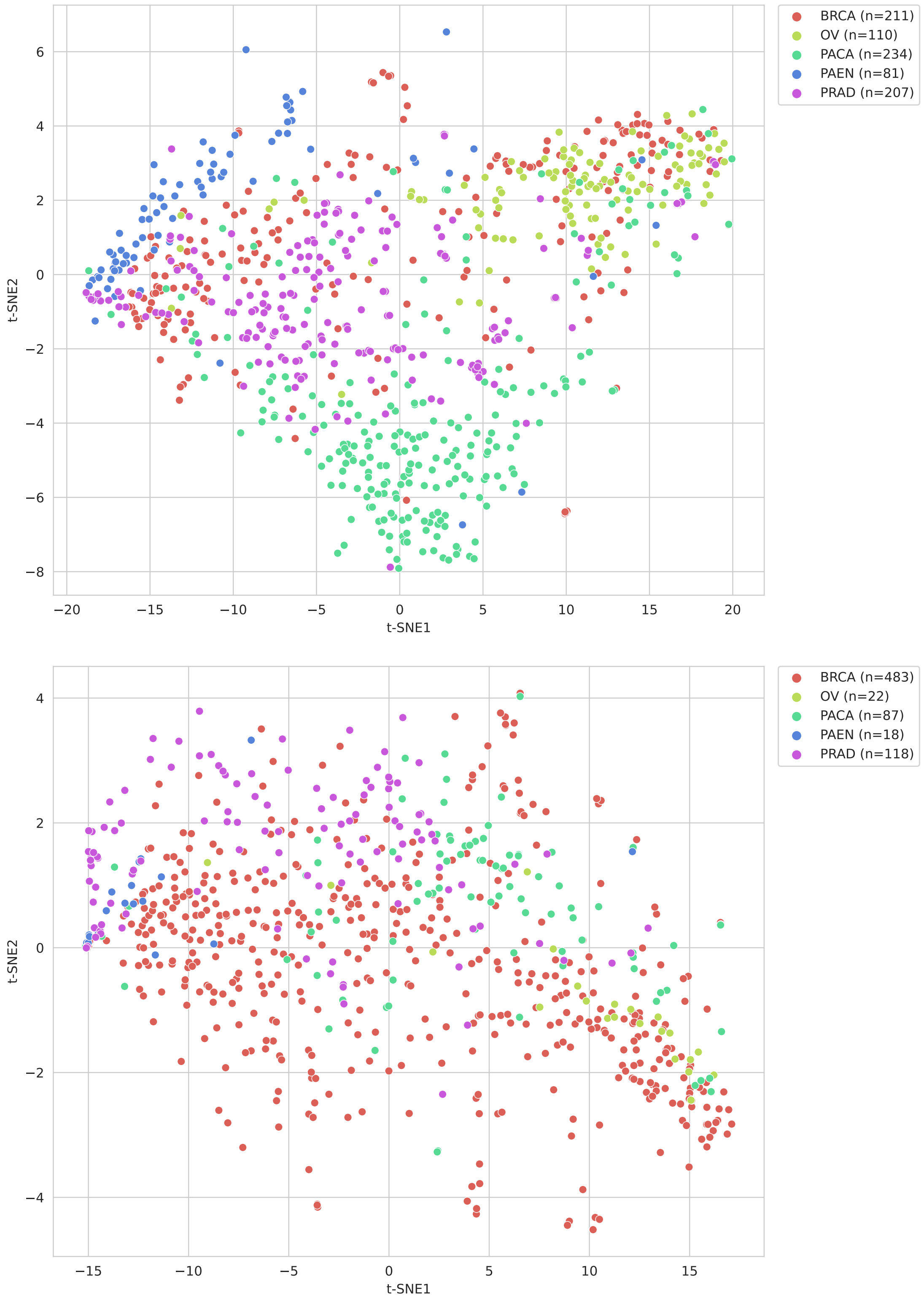

Figure S13: t-SNE plot of cancer mutations data for Breast, Pancreatic, Ovary and Prostate cancer in PCAWG (upper panel) and WGS-extended cohorts (lower panel).

References

[1] Junjun Zhang, Joachim Baran, Anthony Cros, Jonathan M Guberman, Syed Haider, Jack Hsu, Yong Liang, Elena Rivkin, Jianxin Wang, Brett Whitty, et al. International cancer genome consortium data portal—a one-stop shop for cancer genomics data. *Database*, 2011:bar026, 2011.

[2] Serena Nik-Zainal, Helen Davies, Johan Staaf, Manasa Ramakrishna, Dominik Glodzik, Xueqing Zou, Inigo Martincorena, Ludmil B Alexandrov, Sancha Martin, David C Wedge, et al. Landscape of somatic mutations in 560 breast cancer whole-genome sequences. *Nature*, 534(7605):47–54, 2016.
